# Sclareol and linalyl acetate are produced by glandular trichomes through the MEP pathway

**DOI:** 10.1038/s41438-021-00640-w

**Published:** 2021-10-01

**Authors:** Camille Chalvin, Stéphanie Drevensek, Françoise Gilard, Caroline Mauve, Christel Chollet, Halima Morin, Edith Nicol, Eva Héripré, Lucie Kriegshauser, Bertrand Gakière, Michel Dron, Abdelhafid Bendahmane, Adnane Boualem

**Affiliations:** 1 Université Paris-Saclay, CNRS, INRAE, Univ Evry, Institute of Plant Sciences Paris-Saclay (IPS2), 91405 Orsay, France; 2 Molecular Chemistry Laboratory (LCM), UMR 9168, CNRS, Ecole Polytechnique, Institut Polytechnique de Paris, Route de Saclay, 91128 Palaiseau Cedex, France; 3 Laboratory of Mechanics of Soils, Structures and Materials (MSSMAT), UMR 8579, CNRS, Ecole CentraleSupélec, Université Paris-Saclay, Bâtiment Eiffel, 8-10 rue Joliot-Curie, 91190 Gif-Sur-Yvette, France

**Keywords:** Secondary metabolism, Plant physiology

## Abstract

Sclareol, an antifungal specialized metabolite produced by clary sage, *Salvia sclarea*, is the starting plant natural molecule used for the hemisynthesis of the perfume ingredient ambroxide. Sclareol is mainly produced in clary sage flower calyces; however, the cellular localization of the sclareol biosynthesis remains unknown. To elucidate the site of sclareol biosynthesis, we analyzed its spatial distribution in the clary sage calyx epidermis using laser desorption/ionization mass spectrometry imaging (LDI–FTICR-MSI) and investigated the expression profile of sclareol biosynthesis genes in isolated glandular trichomes (GTs). We showed that sclareol specifically accumulates in GTs’ gland cells in which sclareol biosynthesis genes are strongly expressed. We next isolated a glabrous *beardless* mutant and demonstrate that more than 90% of the sclareol is produced by the large capitate GTs. Feeding experiments, using 1-^13^C-glucose, and specific enzyme inhibitors further revealed that the methylerythritol-phosphate (MEP) biosynthetic pathway is the main source of isopentenyl diphosphate (IPP) precursor used for the biosynthesis of sclareol. Our findings demonstrate that sclareol is an MEP-derived diterpene produced by large capitate GTs in clary sage emphasing the role of GTs as biofactories dedicated to the production of specialized metabolites.

## Introduction

Ambergris, a natural perfume ingredient, is a waxy substance secreted by the digestive tract of male sperm whales and the best-known amber odorant natural ingredient^1–3^. Ambergris has been particularly used in the perfume industry for its intense fragrance and unequaled fixative properties^4^. Ambergris properties are mainly due to one of its constitutive components, ambroxide^5^. The growing demand for ambergris-type odorants prompted the search for synthetic alternative routes to ambroxide. The most commonly used route to ambroxide is the hemisynthesis starting from sclareol, natural occurring terpene^6,7^.

Sclareol is a natural diterpene originally characterized in clary sage (*Salvia sclarea*). *Salvia* is the largest genus of the Lamiaceae family of plants with almost 1000 described species^8^. It is widely spread around the world, as *Salvia* species are found in both temperate and subtropical regions. Clary sage is a biennial herbaceous species naturally growing in the Mediterranean basin and Western Asia^9,10^. Due to its remarkable aromatic properties, this plant is grown in different European countries, particularly in France, Hungary, and Bulgaria, but also in North America and China^11,12^. Clary sage has been traditionally used as a medicinal plant^11^ and numerous biological effects including anti-inflammatory, antimicrobial and cytotoxic activity have been associated with its flower extracts^13,14^. Today, clary sage is mainly exploited for its aromatic properties. Essential oils (EOs) and sclareol extracted from clary sage are mainly used in flavor and fragrance industries. EOs are extracted by steam distillation of fresh inflorescences and sclareol is then purified from the remaining plant material by solid/liquid extraction with an organic solvent^15,16^. EO is used directly as a perfume component for its tenacious, herbaceous, sweaty, and amber odor^16^, while sclareol is used as starting material for the hemisynthesis of ambroxide^17^.

In Angiosperms, terpenoid production is often localized in specialized secretory structures^18–22^. The *Lamiaceae* are generally characterized by the presence of epidermal secretory structures called glandular trichomes (GTs), which secrete various compounds mainly involved in pollinator attraction or defense against herbivores^23^. Like most members of the *Lamiaceae* family, clary sage displays two types of GTs: capitate and peltate GTs^24,25^. This classification is based on their distinct morphology: capitate GTs have a long stalk topped by a small spherical secretory head, while peltate GTs are sessile trichomes with a very short stalk and a large flattened glandular head (Fig. S1). Two subtypes of capitate GTs have been described in clary sage according to their size: small and large capitate GTs^25^ (Fig. S1). Linalyl acetate, the acetylated derivative of the monoterpene linalool, is a major component of clary sage EO and is generally considered to be produced by clary sage GTs^11,25^. By contrast, the localization of sclareol production is less clear. A study using a set of microscopy and analytical chemistry approaches highlighted the presence of abundant epicuticular sclareol crystals at the surface of the calyx^15^. The authors of this study hypothesized that sclareol may be produced by calyx epidermal cells, like other cuticular secretions^15^.

Terpene biosynthesis starts with the condensation of five-carbon building blocks: isopentenyl diphosphate (IPP) and its isomer dimethylallyl diphosphate (DMAPP), to form linear compounds called prenyl intermediates. Prenyl intermediates are then converted into functional terpenes through a number of chemical modifications, for example, cyclization and hydroxylation^26^. Clary sage diterpene synthases responsible for the production of the diterpene sclareol from geranylgeranyl diphosphate (GGPP) have been identified by three different research groups^12,27,28^. A class II diterpene synthase was shown to catalyze in vitro the conversion of GGPP into a bicyclic intermediate, labda-13-en-8-ol diphosphate (LPP)^12,27,28^. Besides, a class I diterpene synthase was shown to produce sclareol from LPP in vitro^12,27^. When combined in vitro or co-expressed in yeast, these two enzymes catalyze the synthesis of sclareol from GGPP, in two steps^12,27^.

Genes involved in the biosynthesis of linalyl acetate in clary sage have not been identified so far, but the biosynthesis of this monoterpene has been investigated in several mint species^29,30^. In lemon mint (*Mentha aquatica* var. *citrata*, Lamiaceae), linalyl acetate is obtained after acetylation of the monoterpene linalool, which is produced in one step from GPP through the action of a linalool-synthase^30^.

In plants, IPP and DMAPP are produced by two distinct metabolic pathways: the mevalonate pathway (MVA pathway) and the methylerythritol-phosphate pathway (MEP pathway)^31,32^. As the MVA and MEP pathways are localized in different compartments of the plant cell, cytosol, and plastids, respectively, their respective activities generate two separate pools of IPP and DMAPP (Fig. S2). In agreement with the subcellular localization of the corresponding enzymes, the biosynthesis of downstream products generally depends on only one of these two pools^33^. Most sesquiterpenoids originate from the MVA pathway, while the majority of mono- and diterpenoids derive from the MEP pathway^34^. However, a growing number of studies describe the production of terpenoids of mixed origin, i.e., made of five-carbon units originating from both pathways^33,35,36^. Indeed, prenyl intermediates can be exchanged between the two compartments thereby enabling the connection between the MVA and MEP pathways^37^. The metabolic origin of sclareol and linalyl acetate in clary sage has not been investigated in this context.

To elucidate the site of sclareol and linalyl acetate biosynthesis, we analyzed the spatial distribution of sclareol and linalyl acetate at the surface of the clary sage calyx, using Laser desorption/ionization (LDI) mass spectrometry imaging (MSI). In addition, we investigated the expression of sclareol biosynthesis genes in isolated GTs. Our data show that sclareol and linalyl acetate are mainly produced in GTs. Pathway-specific labeling and inhibitors studies further indicate that the MEP pathway is the main channel for the production of IPP and DMAPP leading to sclareol and linalyl acetate biosynthesis. Finally, we isolated and characterized a glabrous clary sage line to genetically validate the role of GTs in sclareol and linalyl acetate biosynthesis and storage. Our findings demonstrate that most of the sclareol and linalyl acetate found in clary sage is produced by the large capitate GTs, providing new insights in the processes involved in terpene biosynthesis and accumulation in plants.

## Results

### Sclareol and linalyl acetate are highly abundant terpenes of clary sage calyx surface

Studies indicate that the chemical composition of clary sage EO shows important variations and chemotypes have been identified according to the proportion of each compound in the EO^14^. To characterize the chemical composition of the Vatican White variety of clary sage, we performed gas chromatography–mass spectrometry (GC–MS) analysis of a fully developed calyx extract. Two main metabolites were eluted at 6.20 and 16.98 min, respectively (Fig. S3). The electron ionization (EI)–MS mass spectra obtained from the peak at 6.20 and 16.98 min were attributed to linalyl acetate (*m/z* 196.14633) and sclareol (*m/z* 308.27153), respectively (Fig. S3). To provide a further atomic-resolution structural characterization of the clary sage calyx extract, we performed ^13^C-NMR analyses. As for the GC–MS analysis, ^13^C-NMR results show that linalyl acetate and sclareol are the main metabolites in the clary sage calyx extract (Fig. S4).

### Sclareol biosynthesis is regulated during calyx development

**Fig. 1:**
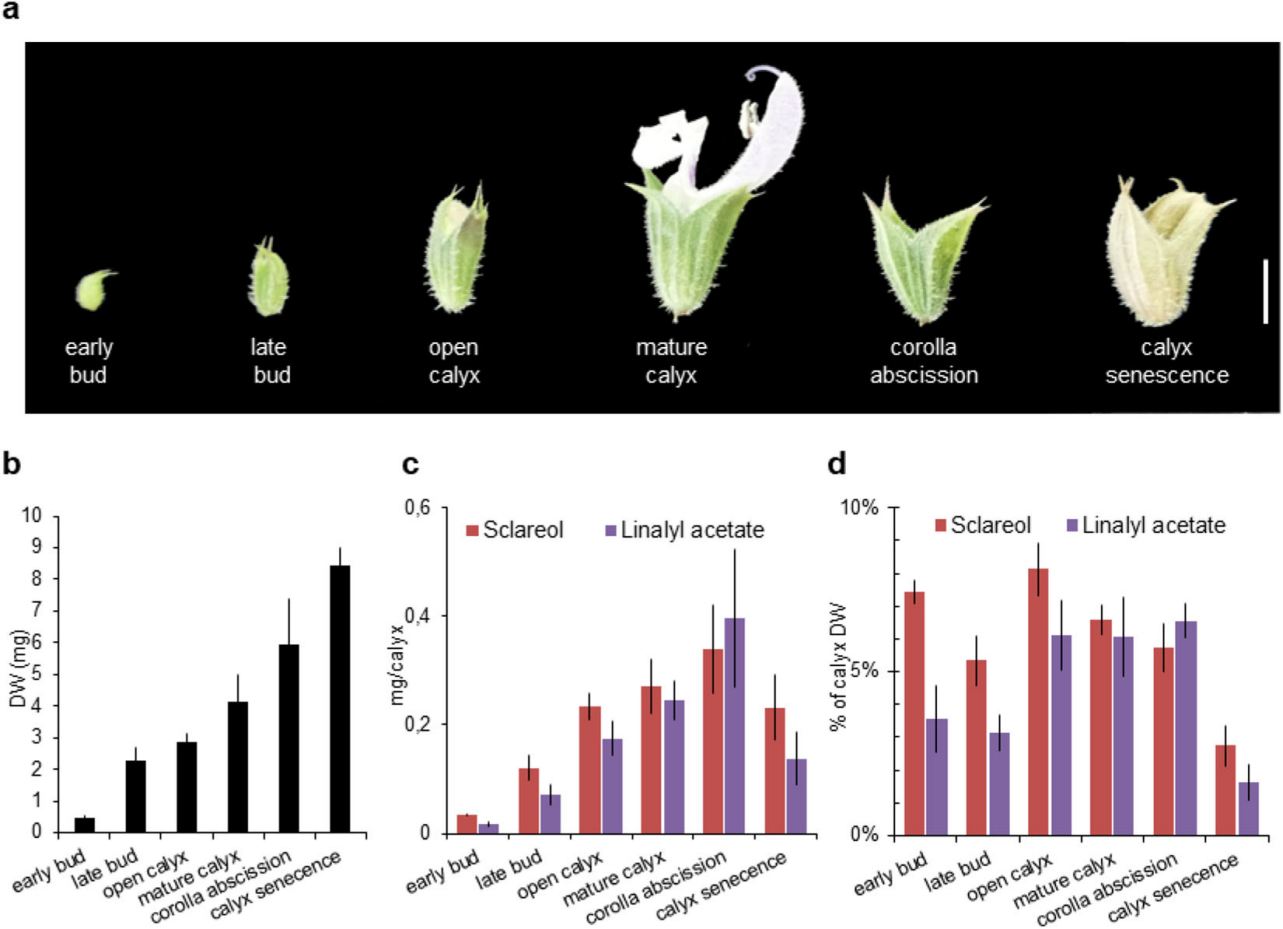
Sclareol and linalyl acetate accumulation during calyx development. **a** Clary sage flower development stages. **b** Flower dry weight was measured after hexane extraction. Hexane extraction was performed without removing the developing corolla from the early bud and late bud stages. **c** Sclareol and linalyl acetate content at different calyx developmental stages. **d** Sclareol and linalyl acetate content expressed as a percentage of calyx dry weight. Bars represent the mean ± SD (*n* = 5). Scale bar: 5 mm

Clary sage calyx is the main production site of linalyl acetate and sclareol^15^; however the production dynamic of these two metabolites, during calyx development, has not been investigated. To explore their production kinetic, linalyl acetate and sclareol were quantified by GC–MS at different stages of calyx development (Fig. 1a, b). We found sclareol and linalyl acetate accumulation starting at the early bud stage, where they represent 7.5% and 4.5% of calyx dry mass, respectively (Fig. 1a–d). Then, the accumulation of sclareol and linalyl acetate gradually increases, reaching a maximum at the corolla abscission stage (Fig. 1c). We found sclareol and linalyl acetate, at the corolla abscission stage, present in comparable amount at ~0.4 mg/calyx (Fig. 1c), making ~6% of calyx dry mass (Fig. 1d). At the calyx senescence stage we observed a significant decrease in sclareol and linalyl acetate content (Fig. 1c, d). The observed decrease in linalyl acetate content could be explained by the evaporation of this volatile molecule^25^. However, the decrease in the concentration of sclareol is unlikely due to evaporation, given its very low vapor pressure (0.000435 mmHg at 25 °C) and that it was not absorbed in clary sage headspace experiments^25^.

### Sclareol and linalyl acetate biosynthesis is inhibited by FOS in a dose-dependent manner

In plants, the isoprenoid precursors IPP and DMAPP are produced by the cytosolic MVA and plastidial MEP pathways^31,32^ (Fig. S2). To investigate the contribution of MVA and MEP pathways to sclareol and linalyl acetate biosynthesis, we used specific inhibitors of each pathway, mevinolin (MEV) and fosmidomycin (FOS). MEV is a competitive inhibitor of 3-hydroxy-3-methylglutaryl (HMG) coenzyme A (CoA) reductase (HMGR), a rate-limiting enzyme of the MVA pathway that catalyzes the conversion of HMG-CoA to mevalonate (MVA)^38^. FOS competitively inhibits the 1-deoxy-d-xylulose 5-phosphate reductoisomerase (DXR), a key regulatory enzyme in the MEP pathway that catalyzes the conversion of 1-deoxy-d-xylulose 5-phosphate (DXP) to 2-*C*-methyl-d-erythritol 4-phosphate (MEP)^31^.

**Fig. 2:**
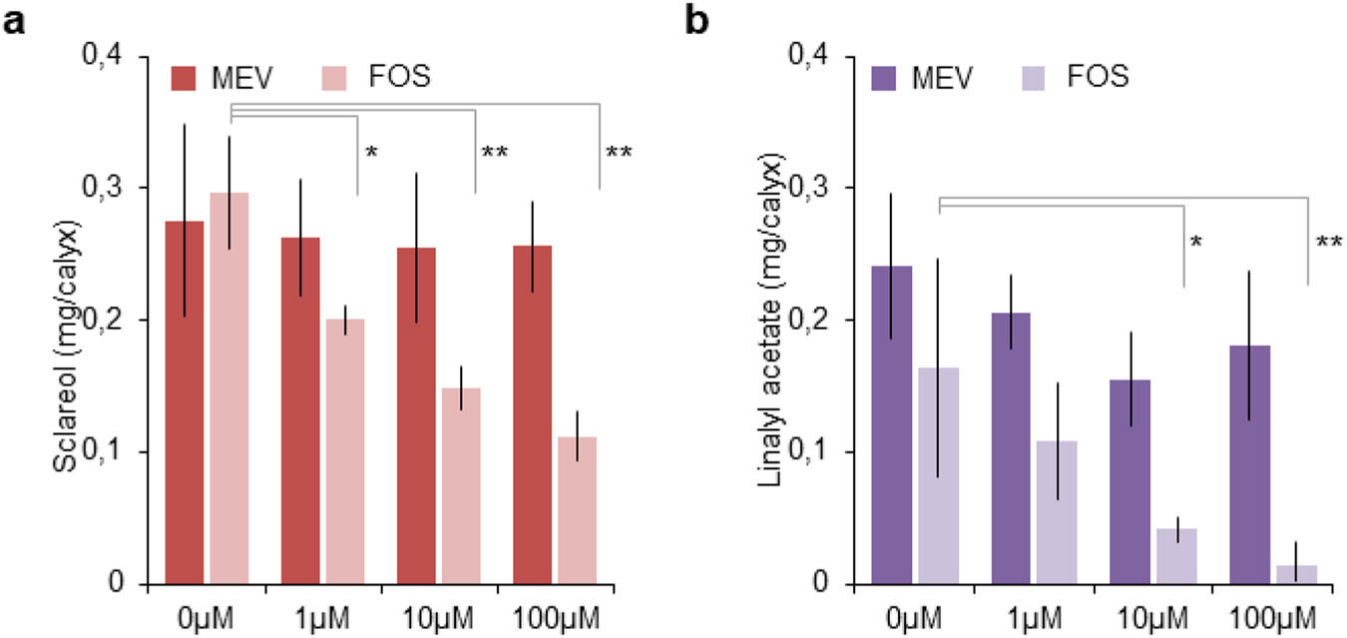
Impact of mevinolin or fosmidomycin treatment on sclareol and linalyl acetate content. Clary sage cut inflorescences were fed with a solution containing mevinolin or fosmidomycin for 6 days. Sclareol (**a**) and linalyl acetate (**b**) content was quantified by gas chromatography coupled to mass spectrometry (GC–MS) after hexane extraction. Bars represent the mean ± SD (*n* = 4). Stars indicate the results of the Student’s *t* test (*p < 0.05, **p < 0.01)

To assess the effect of MEV and FOS, clary sage cut inflorescences were fed with different concentrations of inhibitor, and sclareol and linalyl acetate content were quantified by GC–MS after 6 days. As shown in Fig. 2, FOS treatment inhibits sclareol and linalyl acetate production in a dose-dependent way. At 100 µM FOS, sclareol and linaly acetate contents are reduced by 70% and 97%, respectively, compared with control inflorescence fed only with the nutritive solution (Fig. 2a, b). In contrast, MEV treatment had no effect on the linalyl acetate and sclareol contents (Fig. 2a, b). These results strongly suggest that the MEP pathway is the main source of isoprenoid precursors for both the monoterpene linalyl acetate and the diterpene sclareol biosynthesis in clary sage. The results also indicate that the MVA pathway does not contribute to linalyl acetate and sclareol production.

### Sclareol and linalyl acetate are MEP-derived terpenes

To confirm the role of the MEP pathway in linalyl acetate and sclareol biosynthesis, clary sage cut inflorescences were fed with the stable isotope tracer 1-^13^C-glucose. Sclareol and linalyl acetate ^13^C-labeling patterns were then analyzed by ^13^C-NMR. Depending on the isoprenoid precursor biosynthesis routes, the metabolism of 1-^13^C-glucose gives rise to a specific carbon labeling pattern in IPP and DMAPP^33^ (Fig. S5). Theoretical sclareol and linalyl acetate ^13^C-labeling patterns expected in each case (Fig. S5a) were then inferred based on described enzymatic mechanisms^12,27–30^. If sclareol is synthesized through the MEP pathway, carbons at position C-2, −6, −11, and −15 would be specifically labeled, whereas if it derives from the MVA pathway, carbons would be specifically labeled at position C-1, −3, −5, −7, −9, −12, −14, and −18 (Fig. S5b, c). Sclareol carbons C-16, −17, −19, and −20 would be labeled via either the MEP or MVA pathway whereas C-4, −8, −10, and −13 carbons would never be labeled. Thanks to the high resolution of the NMR spectra of sclareol and linalyl acetate, signals of carbon atoms have been assigned unambiguously to all the positions C-1 to C-20 for sclareol and C-1 to C-12 for linalyl acetate (Fig. S4).

**Fig. 3:**
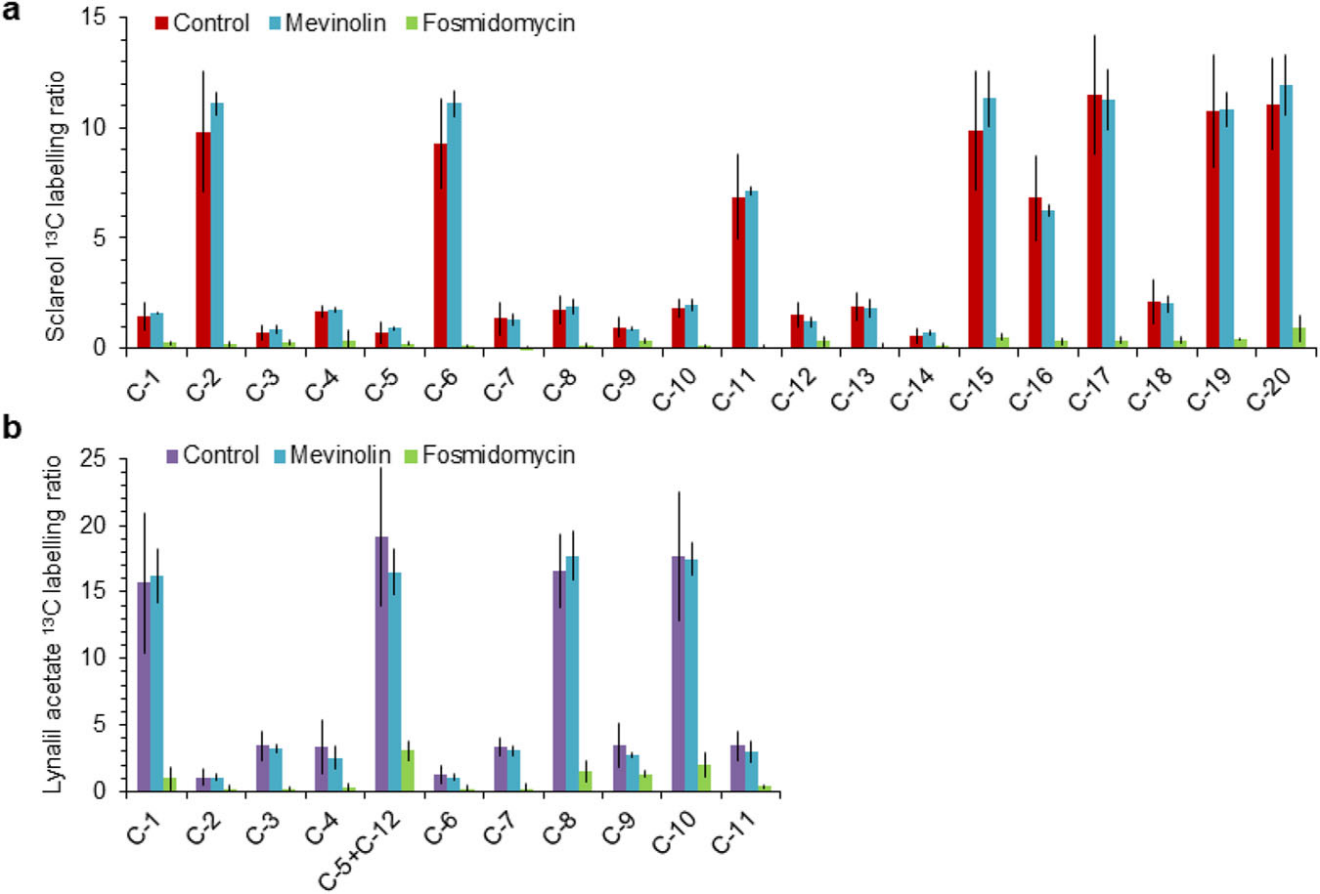
Sclareol and linalyl acetate ^13^C-labeling after feeding with 1-^13^C-glucose and terpene biosynthesis inhibitors. Clary sage cut inflorescence stems were fed with a solution containing 1-^13^C-glucose or standard glucose with 100 µM of mevinolin, 100 µM of fosmidomycin, or no inhibitor (control) for 5 days. Sclareol (**a**) and linalyl acetate (**b**) ^13^C-labeling was analyzed by quantitative ^13^C nuclear magnetic resonance (^13^C-NMR) after hexane extraction. The labeling ratio was calculated as follows: (Peak area_labeled glucose_ − Peak area_standard glucose_)/Peak area_standard glucose_. Bars represent the mean ± SD (*n* = 3)

Consistent with the ^13^C-labeling prediction, ^13^C NMR spectrum of 1-^13^C-glucose fed clary sage shows high ^13^C abundance for C-2, −6, −11, −15, −16, −17, −19, and −20 atoms of sclareol. In contrast, ^13^C abundance for C-1, −3, −4, −5, −7, −8, −9, −10, −12, −13, −14, and −18 carbons was very low (Figs. 3a and S3). Similarly, for linalyl acetate, high ^13^C abundance was recorded for C-1, −5, −8, −10, and −12 whereas it was very low for C-2, −3, −4, −6, −7, −9, and 11 carbons (Figs. 3b and S3).

To assess the effect of MVA and MEP pathway inhibitors on the sclareol and linalyl acetate ^13^C labeling, MEV and FOS were added to the feeding solution together with 1-^13^C-glucose. FOS almost completely abolishes the ^13^C labeling of all carbons of sclareol and linalyl acetate, whereas the ^13^C labeling observed in presence of MEV is comparable to the control (Fig. 3a, b).

Taken together, the MEV and FOS inhibition data and the 1-^13^C-glucose tracer results strongly support the conclusion that sclareol and linalyl acetate biosynthesis depends on the plastidial IPP and DMAPP produced by the MEP pathway.

### Sclareol and linalyl acetate are spatially localized in GTs

Plant metabolomics analyses allow the detection of thousands of metabolites from whole tissues homogenate^39^, but the process destroys and oversimplifies the spatial tissue organization. For example, a leaf epidermis often comprises pavement, guard, trichome, and glandular cells known to have different metabolic profiles. Visualization of the metabolites’ distribution is therefore central to provide cellular and site-specific context for the biosynthesis of target metabolites.

**Fig. 4:**
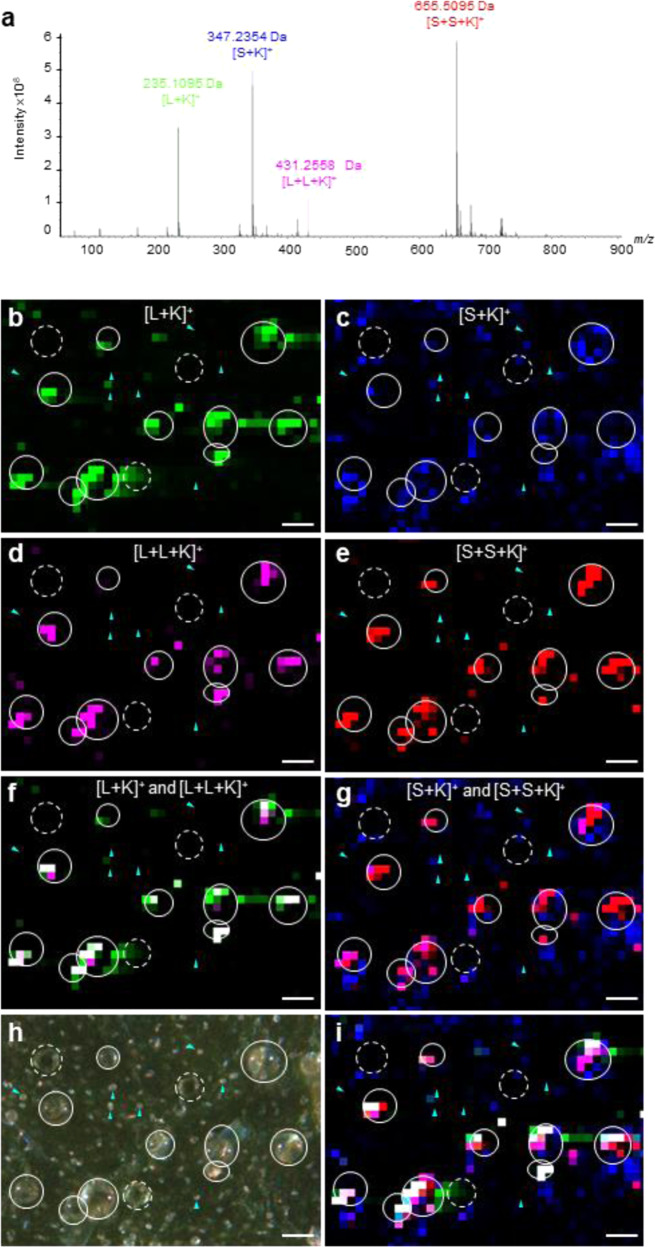
Localization of sclareol and linalyl acetate at the surface of a clary sage calyx. The chemical composition of the epidermal surface of clary sage calyces was analyzed through laser desorption–ionization followed by FT-ICR mass spectrometry (LDI–FT-ICR). **a** Representative mass spectrum of the surface of a clary sage calyx. Spatial distribution of [L+K]^+^ ions (**b**), [L+L+K]^+^ ions (**d**), [S+K]^+^ ions (**c**) [S+S+K]^+^ ions (**e**) and at the surface of the calyx sample. **f** Overlay of [L+K]^+^ and [L+L+K]^+^ ions. **g** Overlay of [S+K]^+^ and [S+S+K]^+^ ions. **h** Top view of a clary sage calyx sample observed with a zoom stereomicroscope. **i** Overlay of [L+K]^+^, [L+L+K]^+^, [S+K]^+^, and [S+S+K]^+^ ions. Blue arrowheads indicate short capitate glandular trichomes, circles indicate large capitate glandular trichomes, dashed circles indicate peltate glandular trichomes. L linalyl acetate, S sclareol, Scale bar: 100 µm

To image and perform in situ metabolic mapping, we use the matrix-free LDI–MSI on fresh clary sage calyx that has not undergone any pretreatment. Ion detection and quantification were performed by Fourier transform ion cyclotronic resonance (FT-ICR), a high-resolution mass spectrometry technique allowing reliable identification of compounds in a complex mixture of chemicals such as found at the surface of plant epidermis^40^. A representative single-pixel mass spectrum from the calyx surface is shown in Fig. 4a. Sclareol (S, *m/z* 308.2715) is detected as a monomer [S+K]^+^ (*m/z* 347.2354, Δ*m/z* = 0.95 ppm) and a dimer potassium adduct [S+S+K]^+^ (*m/z* 655.5095, Δ*m/z* = 2.04 ppm). Dimers ion adducts often form when the molecule is present at a high concentration^41–43^. In line with the GC–MS analysis (Fig. S3), the LDI–FT-ICR spectrum shows that the most abundant ions correspond to sclareol ion adducts indicating that sclareol is highly concentrated in the clary sage calyx (Fig. 4a).

Similarly, linalyl acetate (L, *m/z* 196.14633) is detected as monomer potassium adduct [L+K]^+^ (*m/z* 235.1095, Δ*m/z* = −3.66 ppm). In contrast to sclareol, the dimer potassium adduct [L+L+K]^+^ (*m*/*z* 431.2558) was detected at a very low concentration probably because of the high vacuum applied during the experiment and because linalyl acetate is a volatile monoterpene.

After LDI–MSI spectra acquisition at a pixel size of 25 µm, our data included 6614 metabolite images. Figure 4b–i shows the optical and ion distribution images of sclareol and linalyl acetate at the calyx surface. The spatial distribution of [S+K]^+^, [S+S+K]^+^, [L+K]^+^, and [L+L+K]^+^ exhibits strong similarities. [L+K]^+^, [S+K]^+^, [L+L+K]^+^, and [S+S+K]^+^ ions are observed as intense colocalized spots that correspond to calyx large capitate GTs (Fig. 4b–i). Surprisingly, [S+K]^+^ ion distribution was not exclusively restricted to the area corresponding to the large capitate GTs (Fig. 4c). In contrast, no [S+K]^+^, [S+S+K]^+^, [L+K]^+^, and [L+L+K]^+^ ions have been observed in non-GTs, small capitate and peltate GTs. These results suggest that linalyl acetate and sclareol are most probably biosynthesized in large capitate GTs.

### Diterpene and sclareol biosynthesis genes are expressed in GTs

**Fig. 5:**
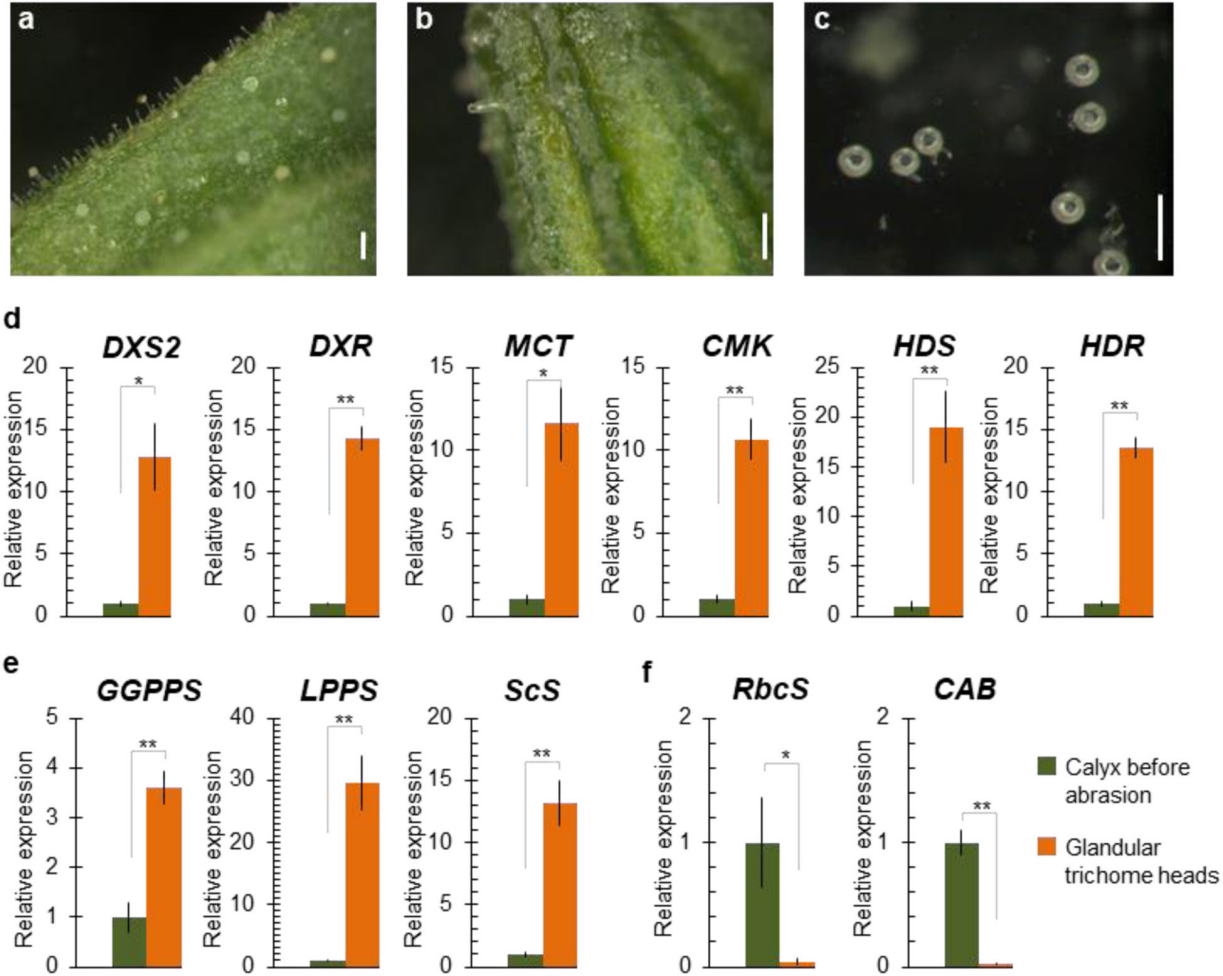
MEP and sclareol biosynthesis genes are expressed in isolated glandular trichome heads. **a**, **b** Clary sage calyx before (**a**) and after (**b**) abrasion of the surface with glass beads. **c** Purified glandular trichome heads. Scale bar: 200 µm. **d**, **e** Relative gene expression of MEP (**d**), sclareol (**e**), and photosynthesis (**f**) genes in calyx before abrasion (dark green) and purified glandular trichome heads (orange). MEP biosynthesis genes: *DXS2* 1-deoxy-d-xylulose-5-phosphate synthase 2, *DXR* 1-deoxy-d-xylulose-5-phosphate reductoisomerase, *MCT* 2-methyl-d-erythritol-4-phosphate cytidylyltransferase, *CMK* 4-diphosphocytidyl-2-methyl-d-erythritol kinase, *HDS* 4-hydroxy-3-methylbut-2-enyl-diphosphate synthase, *HDR* 4-hydroxy-3-methylbut-2-enyl-diphosphate reductase. Sclareol biosynthesis genes: *GGPPS* geranylgeranyl-diphosphate synthase, *LPPS* labda-13-en-8-ol-diphosphate synthase, *ScS* sclareol synthase. Photosynthetic genes. *RbcS* ribulose-bisphosphate carboxylase small chain, *CAB* chlorophyll a-b binding protein. Bars represent the mean ± SD (*n* = 4). Stars indicate the results of Student’s *t* test (*p < 0.05, **p < 0.01)

LDI–FT-ICR imaging alone can only provide conclusive information about the spatial distribution of linalyl acetate and sclareol. To confirm the metabolic imaging results and bring molecular evidence for the metabolite biosynthesis, we assessed the expression of selected genes in GT heads isolated from calyces and in calyces before abrasion (Fig. 5a–c).

Because sclareol and linaly acetate derive from the MEP pathway (Figs. 2 and 3), the expression analysis focused on the MEP biosynthetic genes *DXS2*, *DXR*, *MCT*, *CMK*, *HDS*, and *HDR* (Fig. S2) and genes involved in sclareol production, *GGPPS*, *LPPS*, and *ScS*^12^. The real-time gene expression analysis shows that all MEP and sclareol biosynthetic genes were highly expressed in GT heads compared to calyx before abrasion (Fig. 5d, e). The level of DXS2, DXR, CMS, CMK, HDS, HDR, LPPS, and ScS transcripts is markedly higher in isolated GTs with a 10- to 30-fold relative expression ratio compared to that in calyx before abrasion. *GGPPS* expression is also higher in GTs with a 2.5-fold comparative ratio (Fig. 5e). By contrast, genes encoding proteins involved in photosynthesis, *RbcS* and *CAB*, are predominantly expressed in calyx before abrasion compared to GT heads (Fig. 5f). This observation suggests that there is no or low photosynthetic activity in clary sage GTs is low or absent, as previously reported for mint peltate GTs^44,45^. Taken together, these results confirm that terpenoid biosynthesis, in particular sclareol biosynthesis, mainly occurs in GT heads, in accordance with the data obtained with our LDI–FT-ICR experiments (Fig. 4). Moreover, the fact that the photosynthetic activity is reduced in clary sage GTs suggests that carbon assimilates are transported from neighboring cells to GTs to feed the MEP pathway.

### Large capitate GTs are the main production site of sclareol and linalyl acetate

**Fig. 6:**
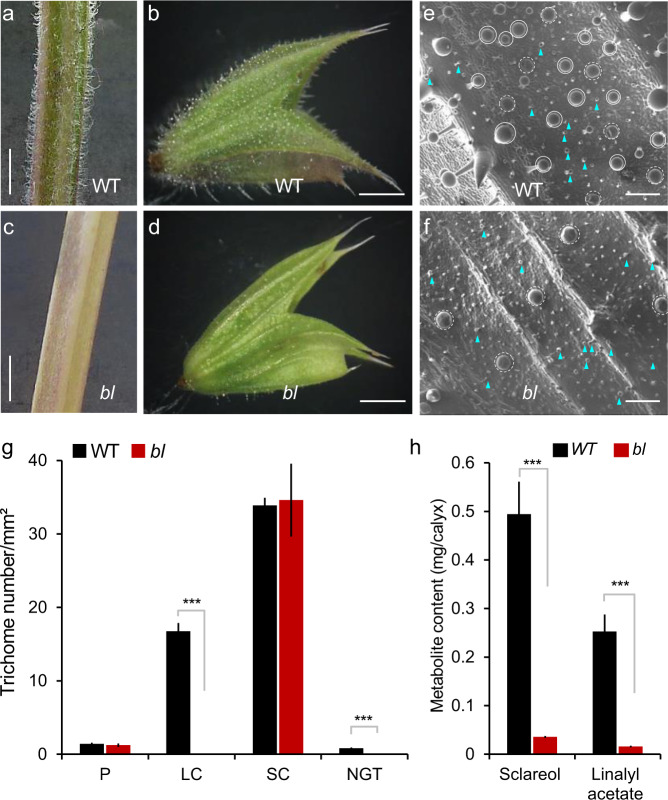
The *beardless* mutant of clary sage displays reduced glandular trichomes density and sclareol and linalyl acetate content. **a**–**d** Phenotype of the wild-type (WT) and *beardless* (*bl*) mutant. **a**, **c** Young stem; **b**, **d** calyx; scale bar: 4 mm. **e**, **f** Scanning electron microscopy of the WT (**e**) and *beardless* (**f**) calyx. Blue arrowheads indicate short capitate glandular trichomes, circles indicate long capitate glandular trichomes, dashed circles indicate peltate glandular trichomes. Scale bar: 300 µm. **g** Density of different trichome types at the surface of calyces from WT and *beardless* plants. Bars represent the mean ± SD (WT, *n* = 9; *beardless* mutant, *n* = 10). P peltate glandular trichomes, LC large capitate glandular trichomes, SC small capitate glandular trichomes, NGT nonglandular trichomes. **h** Sclareol and linalyl acetate content in the WT and beardless mutant plants. Bars represent the mean ± SD (WT, *n* = 18; *beardless* mutant, *n* = 12). Stars indicate the results of the Student’s *t* test (***p < 0.0001)

To provide direct genetic evidence for the role of GTs in the production of sclareol and linaly acetate, we generated a clary sage EMS mutant collection and phenotypically screened 4500 EMS M2 families. We isolated several mutants altered in GT development and density. Among them, the V86 mutant showed a strong glabrous phenotype and was further characterized (Fig. 6a–f).

To assess the inheritance of the glabrous phenotype, the V86 mutant was backcrossed to the clary sage parental line (wild type (WT)) to obtain a BC1F2 segregating population. Among 67 plants, 49 showed a WT GT density and 18 showed a glabrous phenotype in accordance with a 3:1 segregation ratio (WT/glabrous, chi-square test, *p* < 0.01). These results indicate that the glabrous phenotype is controlled by a single recessive gene. We named this locus *beardless* (*bl*).

Scanning electron microscopy imaging of calyces and careful numbering of the different trichome types, GTs and non-GTs, showed that the glabrous phenotype of *bl* mutant plants is due to a complete loss of the large capitate GTs and non-GTs (Fig. 6g). In contrast, the number of peltate and small capitate GTs remains unchanged (Fig. 6g). This result indicates that the development of large capitate GTs and non-GTs share common genetic mechanisms and surprisingly that large capitate, small capitate, and peltate GTs develop through distinct regulatory pathways.

To assess the contribution of large capitate GTs to the clary sage metabolite production, sclareol and linalyl acetate were quantified by GC–MS (Fig. 6h). Metabolite quantification shows that *bl* mutant produces 93% less sclareol and 94% less linalyl acetate compared to non-glabrous WT plants (Fig. 6h). Since the beardless phenotype is linked to the drastic reduction of metabolite production, we concluded that the decrease of sclareol and linalyl acetate content is due to the absence of large capitate GTs, and that large capitate GTs are major sclareol and linalyl acetate production sites.

## Discussion

The epidermis of clary sage calyces develops GTs at high density (Fig. S1). GTs are known to produce specialized metabolites, including flavonoids, acylsugars, and terpenoids^11,23,25,45^. In this study, we provide converging evidence that sclareol and linalyl acetate, respectively a diterpene and a monoterpene, are produced in clary sage calyx GTs. First, [L+K]^+^, [L+L+K]^+^, [S+K]^+^, and [S+S+K]^+^ ions were detected on calyx epidermis by mass spectrometry imaging and coincide with large capitate GTs (Fig. 4). Secondly, gene expression analyses show that genes coding for enzymes involved in the MEP pathway and for diterpene synthases required for sclareol biosynthesis are expressed at high levels in isolated GTs compared to the rest of the calyx (Fig. 5). Lastly, the *beardless (bl)* mutant, a clary sage line with no large capitate GTs, produces 94% less linalyl acetate and 93% less sclareol (Fig. 6). Taken together, these results demonstrate that GTs are the main site of sclareol and linalyl acetate biosynthesis. In particular, large capitate GTs appear to be responsible for the major part of the sclareol and linalyl acetate production in the clary sage calyces.

Peltate and capitate GTs differ in the way they secrete chemicals. Compounds secreted by capitate GTs are regularly released at the plant surface^19,23,46^. In clary sage, scanning electron microscopy observation of a freshly cut mature calyx suggests that capitate GTs secrete their content. Indeed, spills of electron-dense material were visible on many capitate GT stalks (Fig. S6). The use of a pin to puncture a large capitate glandular head confirmed that this electron-dense material comes from capitate GT heads (Fig. S6). The spilling of capitate GT content could explain the [S+K]^+^ ion distribution pattern over the calyx epidermis. Therefore, sclareol present on calyx epidermis, detected in the form of [S+K]^+^ ions, could initially be produced in GT heads and spread over pavement cells along with the rest of glandular head content (Fig. 4). This process of accumulation was shown to occur in the case of the diterpene duvatrienediol in *Nicotiana tabacum*^47^.

Our scanning electron microscopy observations (Figs. S1 and S6) do not confirm the presence of sclareol crystals described by Caissard et al.^15^ at the surface of calyx epidermis. It is important to note that samples used in the study conducted by Caissard et al.^15^ were collected in the field, while our samples were collected on clary sage plants cultivated in the greenhouse. Less than a day after harvest, clary sage calyces collected in the field were covered with crystals, indicating that dehydration facilitates sclareol crystallization^15^. Consistently, we could see sclareol crystals rapidly forming when calyces were heated by a lamp during microscopy observations (Fig. S7). Moreover, sclareol crystals observed by Caissard et al.^15^ were not evenly distributed on the calyx epidermis and were clustered in patches. Similarly, splashes of gland content that we observed by scanning electron microscopy were scattered over calyx epidermis (Figs. S6 and S7). In the field, sclareol crystals may form progressively from splashes of gland content upon dehydration and evaporation of more volatile compounds like linalyl acetate (Fig. S7). This hypothesis was already suggested by Caissard et al.^15^.

Peltate GTs accumulate metabolites under a thick cuticle that ruptures upon mechanical stimulation, for example in contact with an insect^46,48^. They are known to produce volatile monoterpenes and were previously shown to contain sclareol^25,45^. Indeed, it is surprising that no sclareol and linalyl acetate signals could be associated with peltate GTs (Fig. 4). The thick cuticle covering their storage cavity may prevent their content from being ionized upon laser impact^49,50^.

Crosstalk between MVA and MEP pathways occurs when prenyl intermediates accumulate in one of the two corresponding compartments and the excess amount is transferred to the other compartment to supply the other pathway^37^. Such transfers are observed upon treatment with an MVA or MEP pathway-specific inhibitor, but also under particular physiological conditions in specific tissues or at given developmental stages. Notably, transport of prenyl intermediates from plastids to cytosol frequently occurs in photosynthetic organs^26^, whereas transport from cytosol to plastids has been previously suggested to sustain carotenoid and gibberellin production in etiolated seedlings^51^. Interestingly, genes involved in photosynthesis are expressed at low level, if at all, in isolated GTs compared to clary sage calyces before abrasion (Fig. 5), suggesting that the photosynthetic activity of GT cells is low compared to other calyx cells. Therefore, we hypothesized that the MVA pathway could indirectly contribute to sclareol and linalyl acetate biosynthesis in clary sage calyces.

Isotope labeling strategies are powerful tools to study metabolic fluxes and have been successful to characterize the metabolism of many plant species^33^. Notably, the terpenoid-specific patterns obtained with 1-^13^C-glucose labeling are useful to analyze the contribution of the MVA and MEP pathways to the production of metabolites of interest, as was shown with our strategy to assess the biosynthetic origin of sclareol and linalyl acetate (Figs. 2 and 3; Fig. S4). The strong impact of an MEP pathway inhibitor already suggested its involvement in sclareol and linalyl acetate biosynthesis (Fig. 2). However, it could be argued that the drop observed in sclareol and linalyl acetate content is due to a decrease in photosynthetic activity caused by the inhibition of carotenoid synthesis by FOS^33^. The feeding of clary sage inflorescence stems with 1-^13^C-glucose together with FOS rules out this hypothesis and demonstrates that sclareol and linalyl acetate both originate from the MEP pathway (Fig. 3). These results are consistent with the localization of clary sage diterpene synthases involved in sclareol biosynthesis, as these enzymes were shown to be targeted to plastids, the compartment where the MEP pathway is localized^12^.

GTs have been reported to be non-photosynthetic in other Lamiaceae species, including mint^45^. If clary sage GTS are not photosynthetic, as suggested by gene expression analyses (Fig. 5), carbon assimilates must be transported from neighboring cells to GTs to supply MEP pathway activity. A recent study demonstrated that although being photosynthetic, tomato type VI GTs rely on leaf sucrose to maintain their high metabolic activity^52^. This conclusion emerged from the comparison of ^13^C incorporation in primary metabolites of leaves and isolated type VI GTS during ^13^CO_2_ labeling. The same approach could be used in clary sage to analyze the contribution of sucrose from nonglandular cells to the GT metabolism.

Since sclareol and linalyl acetate are mainly produced in GTs, increasing GT density could be a promising approach to increase sclareol and EO production in clary sage. This strategy is currently being developed to enhance the production of the antimalarial sesquiterpene artemisinin in *Artemisia annua*; the overexpression of transcription factors positively regulating GT initiation significantly enhanced GT density and artemisinin yield without any adverse effect on plant growth and fitness^53–55^. Moreover, since sclareol and linalyl acetate are MEP-derived in clary sage calyces (Fig. 5), enhancing MEP pathway activity is another approach to increase sclareol and linalyl acetate content. This strategy has already been implemented successfully to enhance the production of abietane diterpenes in clary sage roots^56^. The ectopic expression of *AtDXS* and *AtDXR* genes in hairy roots led to a 2.2- and 3.3-fold increase in abietane diterpene content, respectively, and some lines overexpressing *AtDXR* even grew faster than the control, thereby combining higher abietane diterpene content and improved growth^56^. Therefore, in addition to improving our understanding of terpenoid production in the Lamiaceae, our results highlight interesting avenues for the targeted genetic enhancement of clary sage performances, which could benefit the flavor and fragrance industry.

## Materials and methods

### Plant material

*S. sclarea* seeds from the “Vatican White” population were purchased from Jelitto (Germany). The *beardless* mutant was generated after seed treatment with ethyl methanesulfonate (EMS). Seeds were immersed in a solution of 0.5% EMS (w/v) for 16 h at room temperature with stirring. Treated seeds were grown in a tunnel and planted as seedlings in the field. Offspring plants were screened for altered phenotypes.

WT and mutant plants were grown in pots for 1 month in a growth chamber (16 h of the day; 27 °C during the day, 21 °C at night; 60% hygrometry) and then 3–6 months in the greenhouse (24 °C from 6 a.m. to 10 p.m., 20 °C from 10 p.m. to 6 a.m.; 60% hygrometry). Pots were moved outdoor at the end of autumn for natural vernalization and left outdoor until flowering. For scanning electron microscopy and LDI–FT-ICR experiments, WT plants were moved back to the greenhouse 2 months before analysis.

### Scanning electron microscopy and trichome numbering

Flower buds (~2 mm long) and mature calyces (harboring a wilted flower) were collected from WT “Vatican White” plants cultivated in the greenhouse (24 °C from 6 a.m. to 10 p.m., 20 °C from 10 p.m. to 6 a.m.; 60% hygrometry). Freshly cut organs were introduced in the observation chamber of a scanning electron microscope (SEM, Helios NanoLab 660 instrument, FEI) without any treatment. Observation of calyx abaxial face (outer face) was performed with the voltage set to 0.35 kV.

For trichome numbering, freshly cut mature calyces were observed with an environmental SEM (MEB SH-1500, Hirox) without metallization or any other sample preparation. Portions of 1.70 mm × 2.45 mm of the abaxial face were scanned at low pressure. The Cell Count Plugin of ImageJ (http://rsb.info.nih.gov/ij) was used to calculate trichome density.

### Metabolite localization by LDI–FT-ICR

Mature calyx samples, harboring wilted petals, were collected from the “Vatican White” variety of clary sage cultivated in a greenhouse. The zone located at the junction of the upper and the lower lip of the calyx, measuring approximately 2 mm × 4 mm, was fixed onto a conductive microscope slide with conductive double-sided tape. Optical images showing top views of calyx samples were taken using a zoom stereomicroscope (AxioZoom V16, Zeiss). Three calyces were then analyzed by mass spectrometry imaging. Matrix-free laser-desorption–ionization was performed using the MALDI source of the SolariX XR 9.4 Tesla FT-ICR mass spectrometer (Bruker). Sampling was performed using the Smart Beam II laser at 25% of its power to avoid sample damage. The distance between the 2 sampling points was set to 25 µm. The spectra were recorded at 120,000 high mass resolution at *m/z* 150 for a mass range of *m/z* 50–1000. Data analysis was performed with the SCiLS Lab 2019b Pro software (Bruker). Data were normalized with the total ion current (TIC). To improve data visualization, for each ion of interest, the 0.3% spots with the highest signal were all displayed with the maximum brightness level using the “hotspot removal” function of the software.

### Identification of the MEP pathway genes

To generate the sequence of DXS2 (DXP-synthase 2), DXR (DXP-reductoisomerase), MCT (MEP-cytidylyltransferase), CMK (CDP-ME kinase), MDS (MEcPP synthase), HDS (HMBPP-synthase), HDR (HMBPP-reductase), and GGPPS (geranylgeranyl diphosphate synthase) genes in *S. sclarea*, nucleotide sequences of the homologous genes were retrieved from *Salvia miltiorrhiza*, *Salvia splendens*, and *Mentha longifolia* genome assemblies^22,57,58^. Nucleotide sequences were aligned and conserved domains were selected to design polymerase chain reaction (PCR) primers. PCR amplification was performed using cDNA library from *S. sclarea* mature calyces. The sequences of the 3′- and 5′-ends of the MEP genes were obtained by Rapid Amplification of cDNA Ends (RACE) using the Marathon^™^ cDNA amplification kit (Clontech). The full-length sequences are listed in Supplementary sequence data.

### GT isolation and gene expression analysis

GTs were isolated from mature calyces as described by Amme et al.^59^ with some modifications. Briefly, flower buds and calyces were collected from “Vatican White” plants, placed in 50 mL Falcon tubes, and frozen in liquid nitrogen. Glass beads of 500–600 µm diameters, purchased from Sigma-Aldrich, were poured on flower buds in presence of liquid nitrogen and GTs were mechanically abraded from the epidermis by shaking with a vortex mixer for 30 s, 10 times. The mixture was filtered through a sieve with a pore size of 200 µm (Sefar) to separate GTs from glass beads and remaining tissue. The purity of GTs was validated by MZ116F stereomicroscopy (Leica).

Total RNAs were isolated using TRIzol (Invitrogen) and treated with DNase I (Thermo Scientific) to remove contaminating DNA. Complementary DNA was synthesized from 1 µg of total RNA using the SuperScript II First-Strand Synthesis System with oligo(dT)20 primers (Invitrogen), according to the manufacturer’s instructions. Quantitative PCR was performed with MESA GREEN qPCR MasterMix Plus (Eurogentech) and 200 nM of each primer in a total volume of 10 μL. Four biological replicates and three technical replicates were analyzed using the CFX384 Real-Time PCR System (Bio-Rad) with the following cycling program: 5 min 95 °C followed by 40 cycles of 15 s at 95 °C and 1 min at 60 °C. Transcript levels were normalized to the transcript level of *ACTIN* and *GAPDH* housekeeping genes. Primers used in this study are listed in Supporting Information, Table S1.

### Treatment with terpene biosynthesis inhibitors

MEV and FOS were purchased from Sigma-Aldrich. Stock solutions (50 mM) were prepared in ethanol and water, respectively. Nutritive solution containing 0, 1, 10, or 100 µM of inhibitor was prepared in 5 mL vials (Murashige and Skoog basal salt mixture (Sigma-Aldrich); glucose 5% w/v (Euromedex); PPM^TM^ 0.1% v/v (Plant Cell Technology); ethanol 0.2% v/v). Clary sage inflorescence axillary stems were cut between the third and the fourth node (about 10 cm from the apex). Each stem was immediately placed in a vial in a way that its extremity was touching the bottom of the vial and dipped in nutritive solution. Treatment was performed by leaving inflorescence stems on the bench for 5 days. Calyces were sampled from each inflorescence stem, immediately frozen in liquid nitrogen, and stored at −80 °C before sclareol and linalyl acetate quantification by GC–MS. Sampled calyces were at the flower bud stage at the beginning of the treatment and were mature at the end of the treatment.

### Isotope labeling and analysis by ^13^C-NMR

Clary sage inflorescence axillary stems were cut and fed with a nutritive solution containing 1-^13^C-glucose 5% w/v (Sigma-Aldrich), following the protocol described above for terpene biosynthesis inhibitor treatment. After 5 days, flower buds and young calyces were sampled from each inflorescence stem, immediately frozen in liquid nitrogen, and stored at −80 °C before further analysis. Calyces collected on one inflorescence stem were immersed in 8 mL of hexane without grinding to extract metabolites present at the surface. After 2 h of agitation, the extract was centrifuged at 13,000 rpm for 10 min to eliminate potential dust. Hexane was evaporated by leaving the samples under the fume hood for 2–3 days. The dry extract was resuspended in 600 µL of CDCl_3_ for ^13^C-NMR analysis. ^13^C-NMR analyses were performed using an Ascend 400 MHz NanoBay instrument equipped with an AVANCE III HD console and a Prodigy BBO cryoprobe (^31^P–^15^N) (Bruker). Analyses were performed at 277 K without tube spinning, using a proton-decoupled (decoupling sequence waltz16) carbon pulse program (zgig) with 90° pulses of 9.75 µs at 41 W for ^13^C. The relaxation delay (D1) was set to 15 s to enable complete acquisition of the signal corresponding to ^13^C nuclei relaxation. To obtain a good signal-to-noise ratio, 5096 scans were launched and data were analyzed using the TopSpin software (Bruker). The attribution of each peak of the spectrum to carbon of sclareol or linalyl acetate (Supporting Information, Table S2) was performed using ^1^H, COSY, HSQC, and HMBC experiments and inspired by previously published data^29,60^. The potential incorporation of ^13^C at a given position was evaluated using the following labeling ratio: (Peak area_feeding_ − Peak area_control_)/Peak area_control_.

### Sclareol and linalyl acetate quantification by GC–MS

Clary sage calyces were immersed in hexane (1.5 mL per calyx) without grinding to extract metabolites mainly present at the surface. 10-undecen-1-ol was employed as internal standard and was added in the solvent before extraction at a concentration of 0.75 mM. After 2 h of agitation, the extract was centrifuged at 13,000 rpm for 10 min to eliminate potential dust. Extracts were directly analyzed using a 7890B/5977A GC–MS system from Agilent, according to a protocol adapted from Laville et al.^16^. The system was equipped with a 10 m guard column and an Rxi-5Sil MS column (length, 30 m; inner diameter, 0.25 mm; film thickness, 0.25 μm) (Restek, Bellefonte, PA, USA). One microlitre of the sample was injected with a split ratio of 30:1. The oven temperature was set to 110 °C for 2 min, then increased to 270 °C at a rate of 10 °C/min, and finally set to 270 °C for 2 min. Other temperatures were set as follows: injector, 270 °C; transfer line, 270 °C; source, 230 °C; quadrupole, 150 °C. The carrier gas was helium at a constant flow of 1 mL/min. The quadrupole mass spectrometer was switched on after a solvent delay of 5 min and was programmed to scan from 35 to 350 u. Data analysis was carried out with the MassHunter Quantitative Analysis software from Agilent. Sclareol and linalyl acetate were identified by comparison with analytical standards purchased from Sigma-Aldrich. Absolute quantification of sclareol and linalyl acetate was performed using a calibration curve prepared with analytical standards.

## Supplementary information

Supplementary Figures Revised

Supplementary Table Revised

## Data Availability

The raw datasets for GC–MS, NMR, and LDI–FTICR experiments are available from the corresponding author on reasonable request. All sequences of *Salvia sclarea* MEP pathway genes generated in this study are available in the Supplementary information (Supplementary sequence data).
