# Supplementary material for: Sclareol and linalyl acetate are produced by glandular trichomes through the MEP pathway
Source: Hortic Res. 2021 Oct 1;8:206. doi: 10.1038/s41438-021-00640-w (PMC8484277; doi:10.1038/s41438-021-00640-w)
Supplement: Supplementary file 1 — Supplementary Figures Revised [file 41438_2021_640_MOESM1_ESM.pdf]

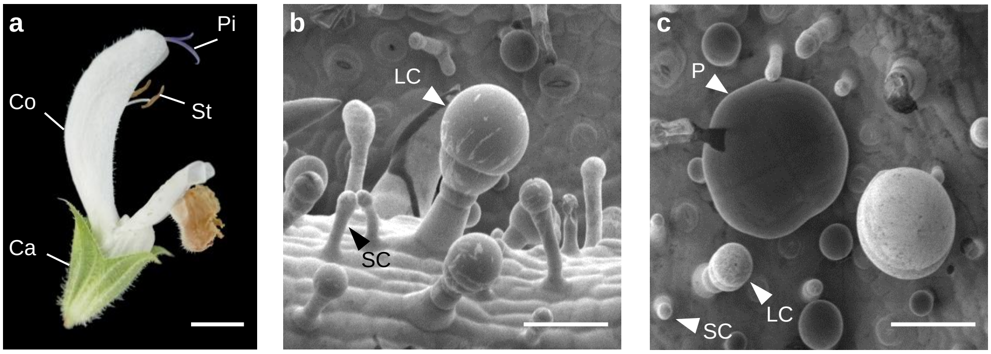

Supplementary Figure S1. Clary sage flower and glandular trichomes. a, Different organs of the clary sage flower. Ca, calyx; Co, corolla; Pi, pistil; St, stamen. Scale bar: 5 mm. **b,c,** Scanning electron micrograph of the surface of a clary sage calyx. Different types of glandular trichomes are observed: LC, large capitate glandular trichome; SC, small capitate glandular trichome; P, peltate glandular trichome. Scale bars: 50 µm

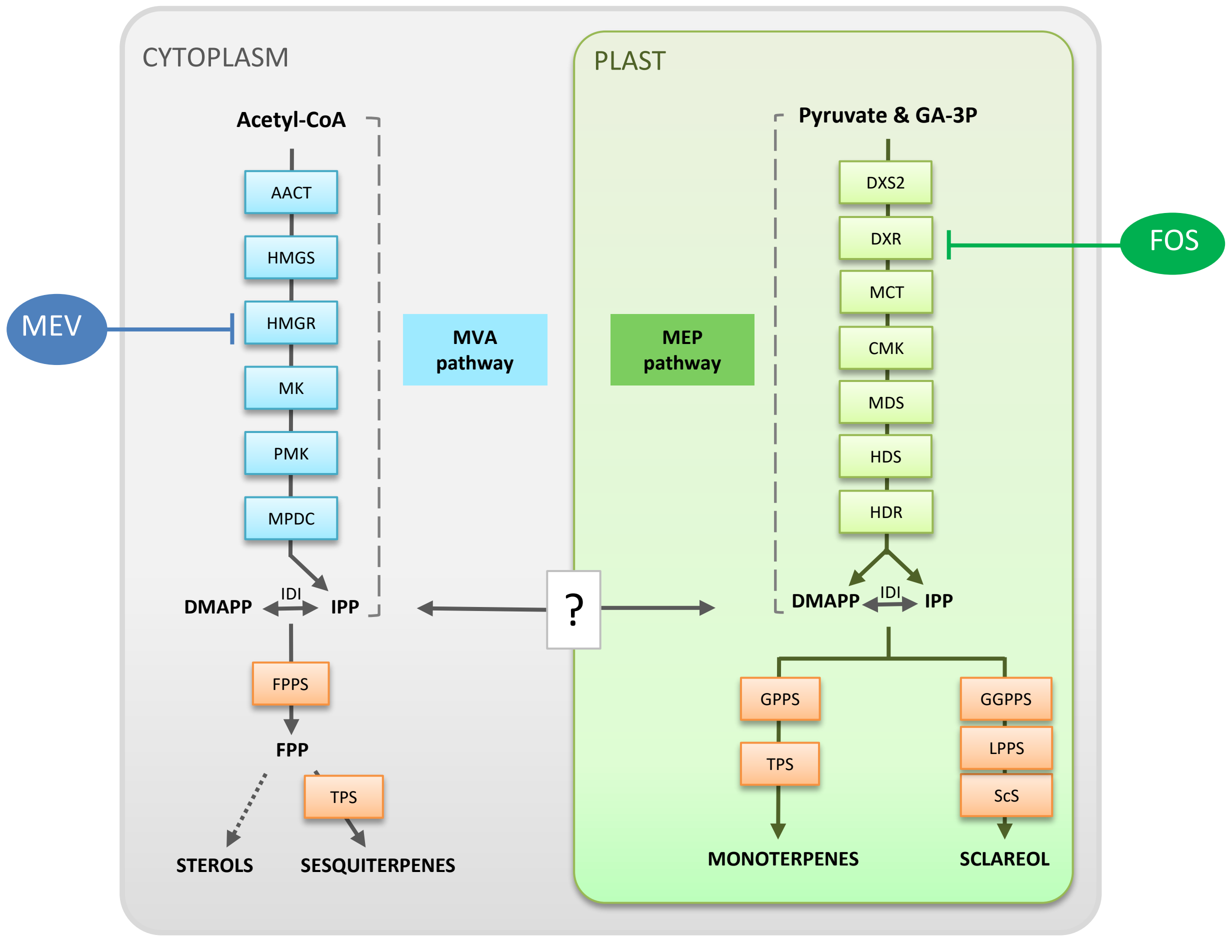

Supplementary Figure S2. Schematic representation of the terpene synthesis pathway in plant cell.

Blue boxes represent MVA (mevalonate) pathway enzymes: AACT (acetoacetyl-CoA thiolase), HMGS (HMG-CoA synthase), HMGR (HMG-CoA reductase), MK (mevalonate kinase), PMK (phospho-mevalonate kinase), MPDC (mevalonate-diphosphate decarboxylase). Green boxes represent MEP (2-methyl-D-erythritol-4-phosphate) pathway enzymes: DXS2 (DXP-synthase 2), DXR (DXP-reductoisomerase), MCT (MEP-cytidylyltransferase), CMK (CDP-ME kinase), MDS (MEcPP synthase), HDS (HMBPP-synthase), HDR (HMBPP-reductase). Interconversion of isopentenyl diphosphate (IPP) and dimethylallyl diphosphate (DMAPP) is catalysed by IDI (isopentenyl diphosphate isomerase). Orange boxes represent enzymes involved in the biosynthesis of terpenes from DMAPP/IPP: FPPS (farnesyl diphosphate synthase), TPS (terpene synthase), GPPS (geranyl diphosphate synthase), GGPPS (geranylgeranyl diphosphate synthase), LPPS (labda-13-en-8-ol diphosphate synthase) and ScS (sclareol synthase). MEV (mevinolin) is a competitive inhibitor of HMGR, a rate-limiting enzyme of the MVA pathway, while FOS (fosmidomycin) competitively inhibits DXR, a key enzyme of the MEP pathway.

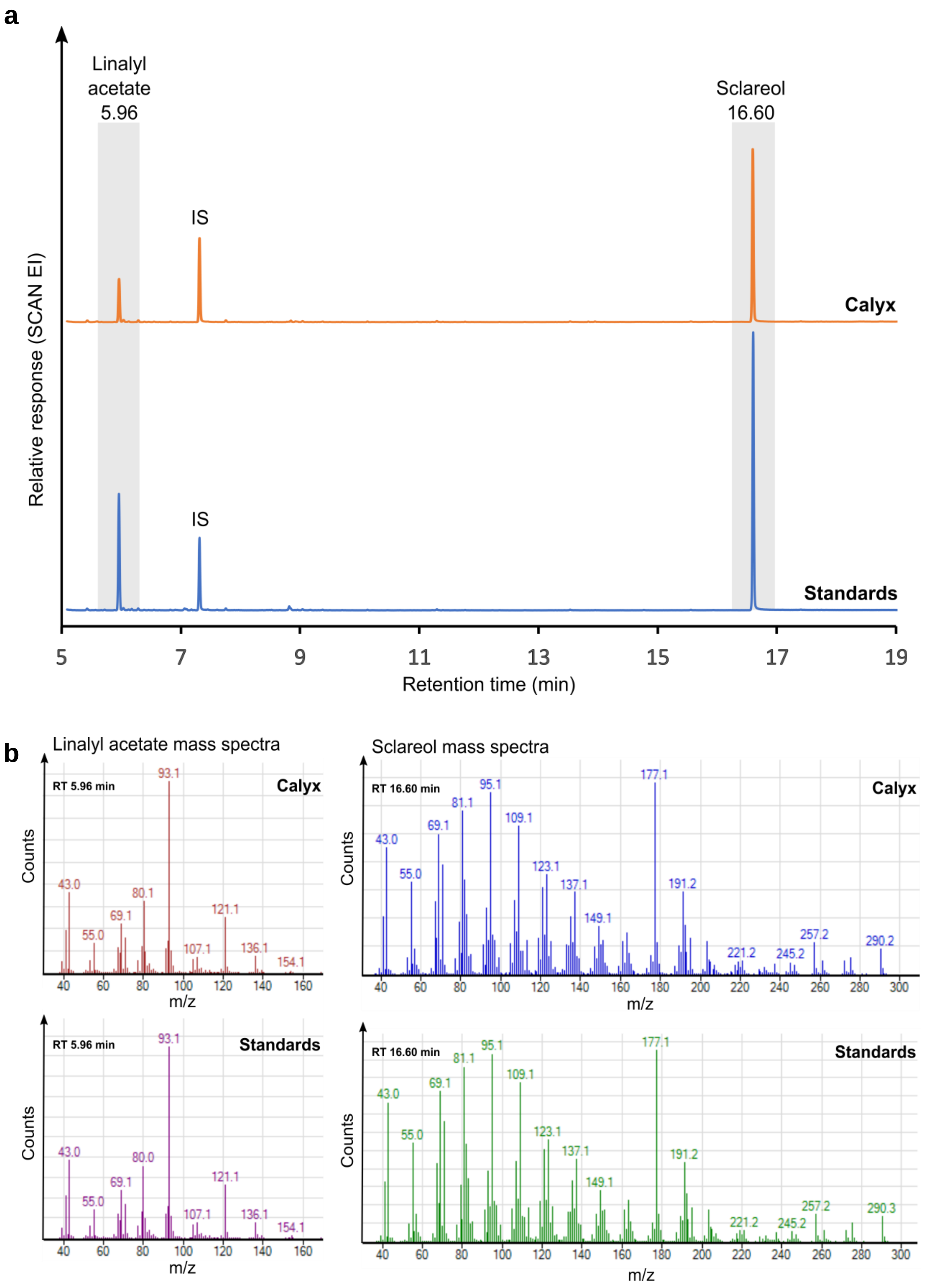

Supplementary Figure S3 : Metabolomic identification of *Salvia sclarea* calyx extracts by GC-MS (EI).
a, Chromatograms resulting of gas chromatography analysis by SCAN of *Salvia sclarea* mature calyx extracts compared with authentic standards. IS: internal standard (10-undecen-1-ol). **b**, Fragmentation spectra of authentic standards and metabolite at the same retention time.

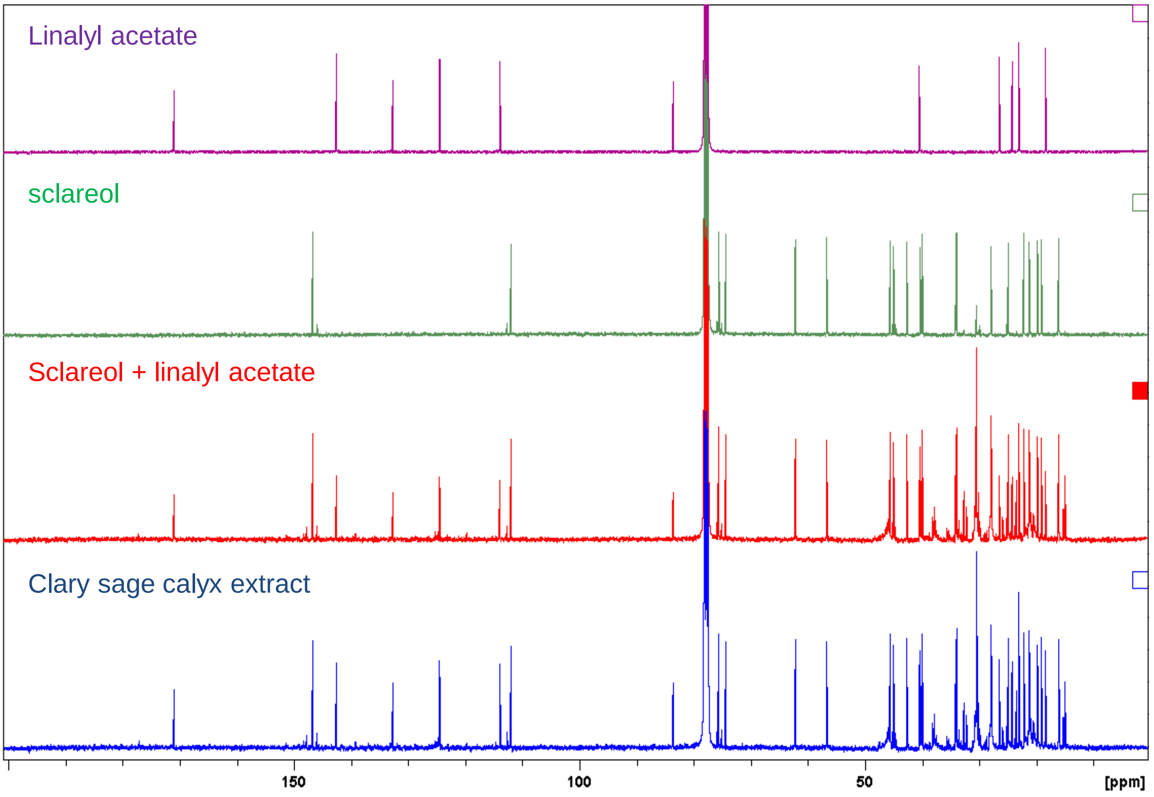

Supplementary Figure S4. Analysis of the composition of a clary sage calyx extract by ^{13}C-NMR.

^{13}C-NMR spectra of linalyl acetate, sclareol analytical standards dissolved in $CDCl_3$ at a concentration of 20 mM. The spectrum in red is a merge of linalyl acetate and sclareol spectra. ^{13}C-NMR spectrum of a clary sage calyx extract obtained after hexane extraction is shown in blue.

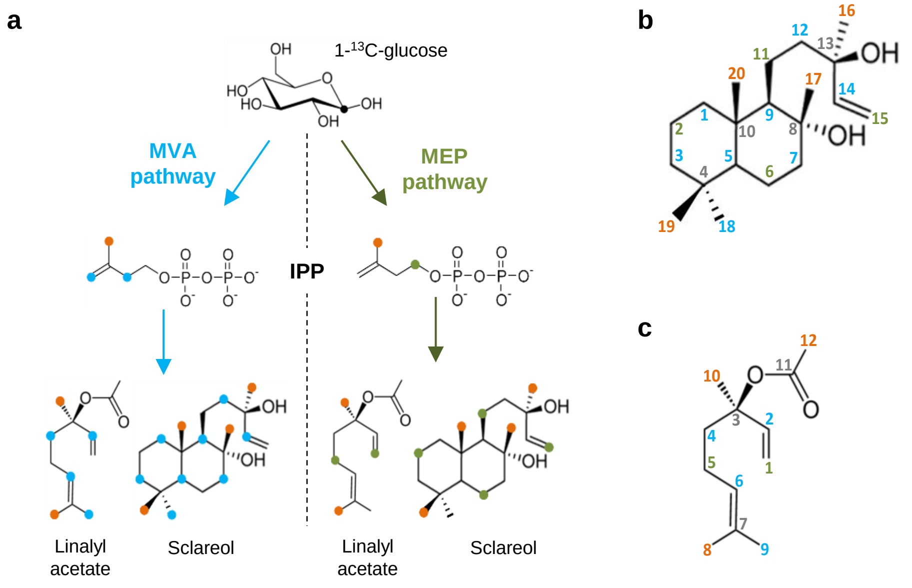

Supplementary Figure S5. Theoretical ^{13}C-labeling patterns of sclareol and linalyl acetate after 1-^{13}C-glucose processing through MVA and MEP pathways.

a, Theoretical positioning of labeled carbons of the intermediates and final products during the synthesis of sclareol and linalyl acetate, according to the pathway used for the synthesis of IPP. **b,c,** Carbon numbering and theoretical position of ^{13}C labeling of sclareol (**b**) and linalyl acetate (**c**). In grey, carbons predicted to be unlabeled; in blue, carbons predicted to be labeled only if the MVA pathway is involved in IPP biosynthesis; in green, carbons predicted to be labeled only if the MEP pathway is involved in IPP biosynthesis; in orange, carbons predicted to be labeled whatever the pathway involved in IPP biosynthesis.

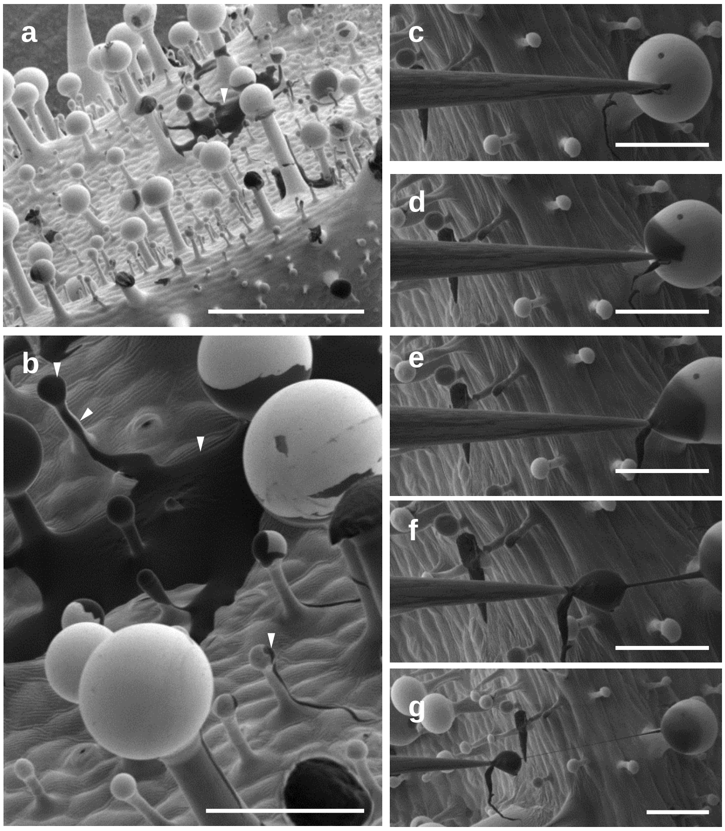

Supplementary Figure S6: Epidermal surface of a clary sage calyx observed by scanning electron microscopy.

a,b, Scanning electron microscopy observation of a freshly cut mature calyx showing spills of electron- dense material (arrow heads) on many capitate GT stalks and calyx surface. **c-g**, Micro-pin puncture of glandular head. **c**, Before puncture, the gland of the capitate GT do not release any content. **d-g**, Once punctured, the glandular head of GT releases electron-dense content that strongly resembles the spills observed on GT stalks and calyx surface.

Scale bars: **a**, 500 µm; **b-g**, 100 µm.

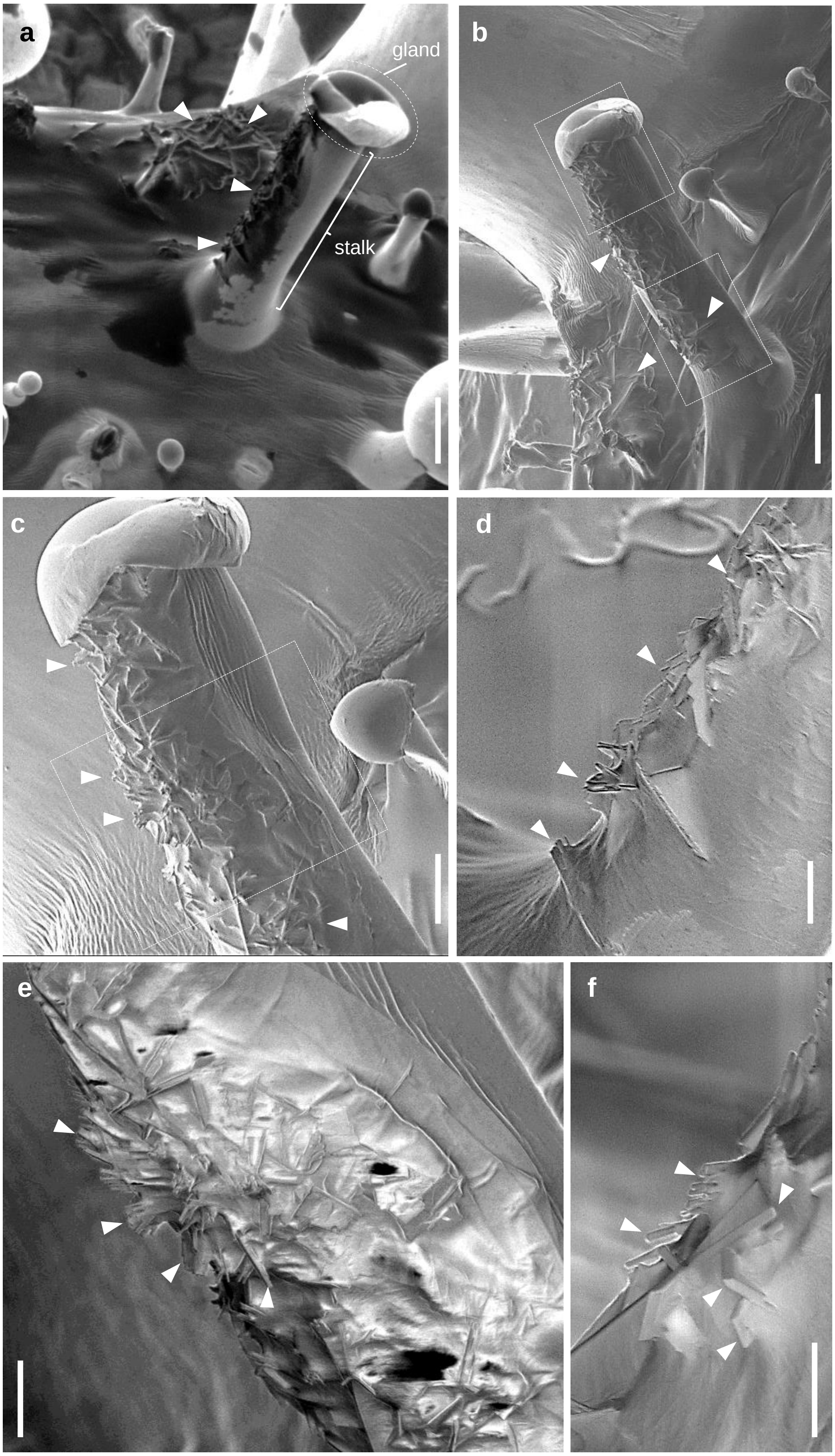

Supplementary Figure S7: Scanning electron microscopy of sclareol crystals formation on GTs.

a,b, Spills of GT content starting crystallization. **c,d,** Closer views of the upper (**c**) and lower (**d**) parts of GT stalk showing crystals formation. **e,f**, Magnified pictures of GT region selected in (**c**). Arrow heads indicate crystal_like structures. Scale bars: **a,b**, 50 µm; **c**, 20 µm; **d,e**, 10 µm; **f**, 5µm.
