# Supplementary material for: Sclareol and linalyl acetate are produced by glandular trichomes through the MEP pathway
Source: Hortic Res. 2021 Oct 1;8:206. doi: 10.1038/s41438-021-00640-w (PMC8484277; doi:10.1038/s41438-021-00640-w)
Supplement: Supplementary file 2 — Supplementary Table Revised [file 41438_2021_640_MOESM2_ESM.docx]

**Supplementary Table 1:** Primers used in this study

| **Primer name** | **Sequence (5' to 3')** |  |
| --- | --- | --- |
| *ACTIN_F* | GAGCCACCACTGAGGACAAT | This study |
| *ACTIN_R* | GGATGGAAGCTGCTGGTATT |  |
| *GAPDH_F* | TCCACTCTATCACTGCGACC | This study |
| *GAPDH_R* | GATTCCTCCTTGATCGCTGC |  |
| *DXS2_F* | GCAGTTTCTTGCCATTGCTCC | This study |
| *DXS2_R* | AGACTCTCTCCACCACTTGC |  |
| *DXR_F* | CGACTGCGTTTGCCTATTCT | This study |
| *DXR_R* | ATTGTCGGGCTTCTTGAATG |  |
| *MCT_F* | GCCATCCTTCAGAACCTTTG | This study |
| *MCT_5* | TGGGAAAGAGAGGCAAGATT |  |
| *CMK_F* | CGAGAGGTACAGGTGGAGGA | This study |
| *CMK_R* | CAAGAGTGGTCGGGTGAGAT |  |
| *HDS_F* | AGCACCGATAACCAAGTCGT | This study |
| *HDS_R* | CAGCAGAGTTCATGCTGCAA |  |
| *HDR_F* | AGGGGTTTGGGCATAAAGAG | This study |
| *HDR_R* | CAGCAAGCTTCACCGTAACA |  |
| *GGPPS_F* | GTGGTGGACATCAACTGCAC | This study |
| *GGPPS_R* | AAAATGGCCCCCAAAACTAC |  |
| *RbcS_F* | CAGAGTCTCCTGCATGAAG | This study |
| *RbcS_R* | TTCCTTGAAGAGCTGCTC |  |
| *CAB_F* | CCTCCTCCAAGTCCAAATTC | This study |
| *CAB_R* | CTAGGTTTTCTGGGACTTCC |  |
| *LPPS_F* | GATGGGTGATATCCGACCAC | Schalk et al., 2012 |
| *LPPS_R* | ATTGCTGATTTCTGGCATCC |  |
| *ScS_F* | TCCAAGGATTTCCTGTGACC | Schalk et al., 2012 |
| *ScS_R* | TCGAAGAGTCGTTGTCGTTG |  |

**Supplementary Table S2: Interpretation of calyx extract ^13^C-NMR spectrum: attribution of peaks to carbon positions in sclareol and linalyl acetate.**

**δ**: ^13^C-NMR chemical shift. In **blue**: carbons predicted to be labeled only if the MVA pathway is involved in IPP biosynthesis; in **green**, carbons predicted to be labeled only if the MEP pathway is involved in IPP biosynthesis; in **orange**, carbons predicted to be labeled whatever the pathway involved in IPP biosynthesis; in **grey**, carbons predicted to be unlabeled whatever the pathway involved in IPP biosynthesis.

|  | **Sclareol** | **Linalyl acetate** | **δ [ppm]** |
| --- | --- | --- | --- |
| **Peak 1** |  | **Carbon 11** | 170,89 |
| **Peak 2** | **Carbon 14** |  | 146,50 |
| **Peak 3** |  | **Carbon 6** | 142,51 |
| **Peak 4** |  | **Carbon 7** | 132,66 |
| **Peak 5** |  | **Carbon 2** | 124,44 |
| **Peak 6** |  | **Carbon 1** | 113,86 |
| **Peak 7** | **Carbon 15** |  | 112,11 |
| **Peak 8** |  | **Carbon 3** | 83,58 |
| **Peak 9** | **Carbon 8** |  | 75,62 |
| **Peak 10** | **Carbon 13** |  | 74,60 |
| **Peak 11** | **Carbon 9** |  | 62,20 |
| **Peak 12** | **Carbon 5** |  | 56,69 |
| **Peak 13** | **Carbon 7** |  | 45,51 |
| **Peak 14** | **Carbon 12** |  | 44,83 |
| **Peak 15** | **Carbon 3** |  | 42,63 |
| **Peak 16** |  | **Carbon 4** | 40,55 |
| **Peak 17** | **Carbon 1** |  | 40,24 |
| **Peak 18** | **Carbon 10** |  | 39,86 |
| **Peak 19** | **Carbon 18** |  | 34,20 |
| **Peak 20** | **Carbon 4** |  | 33,83 |
| **Peak 21** | **Carbon 16** |  | 27,90 |
| **Peak 22** |  | **Carbon 9** | 26,51 |
| **Peak 23** | **Carbon 17** |  | 25,00 |
| **Peak 24** |  | **Carbon 10** | 24,16 |
| **Peak 25** |  | **Carbons 5+12** | 23,01 |
| **Peak 26** | **Carbon 19** |  | 22,21 |
| **Peak 27** | **Carbon 11** |  | 21,19 |
| **Peak 28** | **Carbon 6** |  | 19,75 |
| **Peak 29** | **Carbon 2** |  | 19,11 |
| **Peak 30** |  | **Carbon 8** | 18,24 |
| **Peak 31** | **Carbon 20** |  | 16,12 |

**Supplementary sequence data**

>SsDXS2

TGGCAGGCTAATTTGTCATTTTCTGTATGAGAAGTCACCTCTTTGCATTTACCTCTTGGTCTTAAATTTTTGCTATAAAATGCTCCAACAATCCACCTTCTTATTTCCAGCACTAACAACTTCTCCTCATACAACCTCTCTCTCTCTCAACACGCTTTCATTCTCTGCCTGCTTCAACTCACTCCAAAAGAAAGAGATACAGAGAGAGAGAGAGAGAGAGAGAGATGGCGTCGTCTTGTGGAGTTATCAACAGCAGTTTCTTGCCATTGCTCCATTCCGAGGATTCATCAACCTTGTTATCCCGTTCTAGTGCTCTTCTTTCCGTCAAAAAGCATAAGTTCGCCGTGGTAGCAGCTCTTCAACAGGATAACACCAACGACATGGTTGCAAGTGGTGGAGAGAGTCTGACGACGACGAGGCACAAAACAAGAGCTCTGAATTTCACGGGAGAGAAGCCTCCTACACCAATATTGGATACCATCAACTATCCAATCCACATGAAAAACCTCTCTCTCGAGGAACTTGGGAGATTGGCTGATGAATTGAGGGAAGAAATAGTGTACACGGTGTCGAAAACTGGGGGCCATTTAAGTTCAAGCTTAGGTGTGTCGGAGCTGACTGTGGCACTGCACCATGTGTTCAACACACCAGATGATAAGATCATCTGGGATGTGGGTCACCAGGCCTATCCGCACAAAATCTTGACAGGGAGGAGGGCCAGAATGCACACTATCAGGCAGACATTCGGGCTGGCAGGGTTCCCCAAACGAGATGAGAGCGCACACGATGCATTCGGAGCCGGCCACAGCTCCACTAGCATCTCTGCTGGCCTTGGTATGGCGGTCGGGAGGGACCTATTGCACAAAGACAACCACGTCATCTCAGTCATCGGAGACGGTGCCATGACAGCAGGGCAGGCGTATGAGGCGCTCAACAATGCAGGATTCCTCGATTCCAATCTCATCATCGTCTTGAACGACAACAAACAAGTCTCCCTGCCCACGGCCACCGTCGACGGCCCTGCTCCGCCTGTCGGAGCCTTGAGCAAGGCCCTCACGAGGCTGCAAGCCAGCCGGAAATTCCGCCTCCTCCGCGAAGCAGCAAAGGGCATGACTAAGCAGATGGGAGACCAGGCCCATGAGATCGCATCCAAGGTGGACACCTACATGAAGGGGATGATGGGGAAGCCCGGCGCCTCCCTGTTCGAGGAGCTTGGGATTTACTACATCGGCCCCGTCGACGGCCACAACATCGAAGATCTGGTTTACATTTTCAAGAAGGTGAAGGAAATGCCCGCGCCTGGACCTGTTCTGATCCACATCATCACAGAGAAGGGCAAAGGCTACCCTCCCGCAGAAGTCGCCGCAGACAAAATGCACGGCGTGGTCAAGTTCGATCCTACAACGGGAAAGCAGCTGAAGTCGAAAACCAAGACCAAATCATACACACAGTACTTCGCGGAGTCTCTGGTGGCGGAAGCAGAGCAGGACGACAAGATCGTGGCGATCCACGCGGCGATGGGCGGGGGCACGGGGCTCAACTACTTCCAGAAGCGGTTCCCTGACCGGTGCTTCGACGTGGGGATCGCGGAGCAGCACGCGGTCACCTTCGCGGCCGGGCTGGCCACGGAGGGCCTCAAGCCCTTCTGCACGATCTACTCGTCTTTCCTGCAGAGGGGATACGATCAGGTGGTGCACGACGTCGACCTTCAGAAGCTCCCCGTGCGCTTCATGATGGACCGTGCCGGCGTGGTCGGCGCCGACGGCCCCACCCACTGCGGCGCCTTCGACACCACCTACATGGCCTGCCTCCCCAACATGGTCGTCATGGCTCCCTCCGACGAGCTCGAGTTAATGCACATGATCGCCACAGCCGCCGCCATCGACGACCGCCCCAGCTGCGTCCGCTACCCCAGGGGAAACGGCGTCGGCGCGCCGCTCCCGCCTAACAACAAAGGAACTCCTCTCCAGGTTGGGAAGGGAAGGATATTGAGAGAGGGGAGTAGAGTTGCCATTCTAGGGTTTGGAACTATAGTGCAGAACTGTTTGGCGGCGGCGCAGCTTCTCCAAGAGCACGGCGTGTCCGTCACGGTAGCCGACGCCAGATTCTGCAAGCCGCTGGATGGAGATCTGATCAAGAAGTTGGTGCAGGAGCATGAAGTTCTCATCACTGTTGAAGAGGGATCTATTGGTGGATTCAGCGCTCATATTTCTCATTTCTTGTCTCTCAACGGACTTCTCGACGGGAATCTTAAGTGGAGGCCAATGGTTCTTCCCGACAGATACATCGATCATGGGGCACAGACCGATCAGATTGAAGAGGCTGGGTTGAGTCCAAAGCATATTGCAAAGACTGTTGTGTCACTTATTGGTGGAGGAAAAGATAGTCTTCATTTGATCAACAACTTGTAATCTAAATTTCGTCCAAGAAACAACGTTAGTGATGGTGCCGGAACTCGAGCAGCCAGCTGCAGTTGATCTCCTCAGAGATGTTAGTTTTATGATGTAATGTAAATATACATGGGGATCGATTGCTGCTGGAAGACTCTAACCCATGAAGTTGGGGGAGTTTTTCTAAATAATCGTTCAGATGGCAACCTTATGTTTGTAGAAACAAATAATTACTCCATACTTTTATTAAATAAATTCATTTGTCCCATTCATACATTTCTAAGTGCAGTTATGTTCCAAGTTAAGATTATATTTGTGTGTATAGCCATGTGTCAATTTTAGATATTTCCTTCGTCCCCCATATTTATACATCTTTTGCTTTTTGATATATCCCACAAAATTATGCA

>SsDXR

CGATTCCTTATAAAATAGAGCCGAACTGTTATTATTGTACACAATCTACCTCCTCTCTCTCTCTCTCTCTCTCTCTCTCTCTCTAACCGTCACTAATTTGTTTATAGTTTGCCATCCTTGACCAACTCGAAATTACAGTGTTGGTGCCTACTTATATCTCAGGTTTCTTGAAATAAAGAGAGGAAGAGACTGAAAAAGAAAGCACCTTTTTCTCAATCTTCAGCTTTTCTGTGCATTTCAGCTTGTGATAAGCCATGGCTCTCAACTTGATGTCTCCAACTGAAATCAAGACTCTGTCTTTCTTGGATTCCTCCAAATCGAATTACAATCTCAATCCTCTCAAGTTCCAAGGTGGATTTGCTTTCAAGAGGAAGGATAGCAGATGCACTGCTTCAAAGAGAGTCCATTGCTCGGCACAGCCACCTCCTCCTCCGGCTTGGCCCGGGAGGGCTGTTCCTGAGCCCGGTCGTATGACATGGGAGGGCCCGAAGCCCATTTCGGTTATTGGATCCACTGGCTCCATTGGAACTCAGACGTTGGACATAGTTGCTGAAAATCCGGATAAGTTCAGAATCGTGGCACTTTCTGCTGGTTCAAATGTCACTCTGCTTGCTGATCAAGTGAGGGCTTTCAAACCCAAATTAGTATCCGTGAGAGACGAGTCATTAGTTAGTGAGCTCAAAGAGGCTTTGGCTGGTATTGTGGAGATGCCTGAAATTATTCCGGGAGAGCAGGGAATGGTCGAGGTTGCACGCCATCCCGATGCTGTTACTGTAGTCACGGGAATTGTTGGATGTGCTGGTTTGAAGCCGACAGTGGCTGCCATAGAAGCTGGAAAAGACATTGCTTTGGCCAATAAAGAGACACTAATTGCTGGAGGACCTTTTGTCCTTCCTCTTGCAAAGAAGCATAATGTCAAGATTCTTCCTGCGGATTCTGAACATTCTGCTATATTTCAGTGTATCCAAGGCTTGCCAGAAGGTGCTTTGAGGCGTATAATTTTGACCGCATCCGGGGGTGCTTTCAGGGATTTGCCAGTTGAGAAATTGAAAGAAGTGAAAGTAGCAGATGCTTTAAAGCATCCCAACTGGAATATGGGAAAGAAAATTACAGTGGACTCTGCAACCCTCTTCAACAAGGGTCTAGAAGTTATAGAAGCTCACTATTTGTTTGGGGCCGAATATGATGATATCGAGATTGTTATCCATCCTCAATCTATCATTCATTCGATGATTGAAACACAGGATTCTTCCGTGCTAGCGCAATTGGGATGGCCCGACATGCGTTTGCCTATTCTATACACCTTATCGTGGCCGGAGAGAATCTACTGCTCCGAGATTACATGGCCTCGCCTTGACCTTTGCAACGTCGACCTAACATTCAAGAAGCCCGACAATGTCAAATACCCTTCGATGGATCTAGCTTATGCTGCTGGACGAGCTGGAGGCACCATGACCGGAGTTCTCAGCGCAGCCAACGAGAAAGCAGTTGAAATGTTCATCGACGAGAAAATCGGTTACCTCGACATATTCAAGGTTGTGGAGGTGACGTGCGACAAGCACCGAGCGGAGATGGTGTCGTCGCCTTCGTTGGAGGAGATCATCCACTACGACCAGTGGGCCCGGGATTACGCGGCGGGCGTGCAGCGGTCTGCGGGATTGAGTCCTGCTCTTGTATGAGCAGAGGTTGATGGATGTGATCATCAACTGGAAACTTGTTCCATTTCTTTTTCTTGGTTCTGTTTTTCCCTTCTTTGTTTGGGGGGAAGTCATTTATCATGAAAAGGAAAGGAATCATGTGACATTTATGCAACAGTGCCACCATAAAATAGATTCCAAAAAAAAAAAAGGGTCACGTGATTCTGTGTTTTTGATATTCATCATCGAAGTGTAAATTTGATGTCCAATGTTTTTCATAAGTTCCTTTTCTGAAAAGGGCATTTAGTGAGAGGGTGGAATAAATTTTAAAGATACATGAATATAAATTGTGTTTTCCTCATAATTGGGCCAAAAATATCAGAATTAGGACACTAATTGCGGCAGAATATCAATGTGAATTTTTGTATGACAGTGTTCTCGGAGTTTGAATGCATCAAACTAATATTCTTGTGTAAAGTTAATAGTTCGTAGATGGTGTTAATTTTTTACGGTTCTTTATTTATTATTACGAGGGCTGTATACATTACGACTTGGGCTTCAGGTGCTAAAATGT

>SsMCT

CCGAAATTAAACAGAAGCAAAAAATTTGAGCTCCAAGACTGAAATTGAAGGGAAACCAGAGCTCCCTATTGGCTATTGTCGTCGAGTACAATACATCACGGCCATGCTCTATATTTAAATGAAGACAGAGAAAATCCCAACACACTTTTCGTCTCTAAAACTTCCCTTTCATTCATTCATTCATTCATTCATTCTTCTTTCATTCACCGGCTTCACAAGACTCTTTGAGGAAGAGAAGCAAAGATGTCAACACTTCAATACAGAAATCTCCCACCCTCTTCAACTGCTCTCTCTGCTTCCCCTTCTTCTTTCCCGCTGTTTCTCGGTGCAACTTTGGGGCTCCCAACTAAATTCCGCCCCTCATTTGCTTTGACCACCGTATATAACAAAAGAAAGATCGATTTTGTGAAGAGCCCAATATCATTTAGAATTAGTTGCTCTGCGGGGGGTGCAGACACACAAGTAGTGGTCAAAGAGAAGAGTGTTTCCGTTATTCTGCTTGCAGGAGGAAAGGGCAAAAGAATGGGTGCGAGCATGCCTAAGCAGTATCTTCCACTTCTTGGCCAACCGATAGCTTTATACAGTTTCTACACTTTCTCGAAGATGCCTGAGGTGAAGGAAATCATTGTAGTATGTGATCCTTCGTATCGAGACATTTTTGAAGATGCGAAAGAGGATATTCACATTGACCTAAAATTTGCGTTGCCTGGGAAAGAGAGGCAAGATTCTGTATACAGTGGATTAGAGGCAGTTGATTTGAATTCTGAGCTAGTCTGCATACATGATTCTGCAAGACCTCTCGTTTTAACTTCAGATGTCACAAAGGTTCTGAAGGATGGCAAGCGAATCGGAGCAGCTGTGCTAGGTGTTCCTGCTAAGGCTACAATCAAGGAGGCAAATAGCGAGTCTTTTGTGGTAAAAACACTGGACAGGAAGACACTCTGGGAAATGCAAACCCCACAGGTTATCGAGCCAGGCTTGCTTAAGAAAGGTTTTGAGCTTGTTAATAGAGAAGGACTCGAAGTTACCGACGACGTCTCGATAGTGGAGCACCTCAAACATCCAGTGTACATTACTGAAGGATCTTACACCAACATCAAGGTTACCACCCCAGACGATCTGTTGCTTGCTGAGAGAATATTGAACCCTGAAGATTGAGATCCCTTTAACTTGTTTGAATATTTAATTGAAGTCGATGTAGTTGAGGTTGTCATTCACATGCCCAACTTGACTTTTCATTCTAATTTGTATTTTTGTTACTAGTGAAAGGTGCACATCTTGTTGTATGGACAAACAATGATCCCAATTTTGGAAGAATAATAAAATGCATATACGACAGCACTCAACACTCAAGAGGACTGTTATATATAGAGCTCAGCTCATTATGAAAACTCGCCATTTAGATATACTAATAAAGATATCAAAGTAGCCATTGCTTTGAAAAACATATACTCCATTATATCATACTGTATCCTTAATTTAATATAATGAGAGTAACTTAACGAAATAATAAAAGTATTTCTTAATCGCCAGAAAGTTGAGATTCCCTTTTTAATAGGTTTATGAAAGCCAAACCAATAACTTTTTATTATGTGGCAATATTGAAGTTAGAAATATTACTCCATTGATTGAGGCCCAAACAAAGCAAAGCTGTTGAGTGTAAACGATGGCCTTTTGAAGCCCAATCCACTTCCATTAGAGATCTAAACGATCCCCCATATTTTACTCTCTAAACACCTCATTTCTCATTAATCATTTGCTATTTTCCACACACACAGAGAAAGCAGCGTTAATGGCGAAGCCACTGGGAACAACCGGCGAGTTTTTCAGGCGACGTGACGAGTGGCGGAAGCACCCGATGCTCACCAATCAGTGGCGCCACGCCACCCCCGGCCTCGGCATCGCGCTCGTCGCCTTCGGCATTTACCTCGTCGGTGAAACCGCTTACAACAAGATCTACGCCCCAAAATCTCAGTCTCATTCTCATTCCACCTCTTCCGCTCATCACTGAAATCAGCCATCGGTTACCGATGAAAGGGTGGTGTCAAAGAAAGTAGCAATATCCAGCTTCTCACTTTCCTTCATATTCCTGAATGTTTAATAATAATGCAGTGTTGAGACTTGTTTGTTGAATGGCAGTCTTGTCTGCCAAAACTTCAAATTTGTAACAATGTGAAGAATCATATTTTACTTTTTGCGGGTGTGCTTCTGTTTGGTTTCAATACTAAGATTTAATCGCTACGGGCCAATGTAATAAGCAAATTTGGGCTGGTTGAGTTCCGCGCCCAAGGGATCCTCTTTTCCAAAACAAAGCAAAAGAAAAAAAAGGTGAATCAAGTAACAGATGCACAAGTTAGTGTACATGTACAATAAAATTTCACAAACCAATGGAAAATTTTAGTCTTAACTTTTAGTTATAAATAAAAGGGAGGGGGAGTTTAAAGCGTAT

>SsCMK

GAAGAATGGTGCAACCGCAATCAGCAAAATCGCTGACCAATGCAAAAAAAAAAAAAAAAAAAAGAAAAAAAAAAAAAAAAGAGACACTCTCTCTCTCTCTCTGGTCTAAACATAGGCAGGAGCTGGAAATTTATGTCAGTTGAATAAGTTTAAGCAGAGGAAATGAGGAATCTGTAATCTGCCTTCAAAAATTGAATGTTATTTATTCAACATCTAACAAAATAATAGTGTAGCAGTCAGTTTATTTGTGGGCTACTTGCTTCTCTCTCTCCAATTCCAATGGCTGCTTCCTCCCATTTCCTCTGCAGTCACGCTTCCTACAATCCCAAAACACATTTCAATTCATTCACCAGCGCCACTCTCCCTCAGTTTTCCTCGTTTAAGCCACATGGCTCCTCGTCTTTCCGCAAAAAGATTCAGTCTTCAAGAATCCATCTTATCAGAGCCACGGCTTCTGATTCCACCACCGGCAGAAAACAACTAGAGGTGGTATATGATCTTGAGAATAGGTTAAATAAGTTGGCTGATGAAGTGGATAGGGAAGCTGGGCTTTCAAGACTCACTCTTTTTTCGCCTTGCAAGATTAACGTTTTCCTAAGAATAACTGGCAAGCGAGCAGATGGATTCCATGATTTGGCGTCTCTTTTTCATGTTATCAGCCTAGGGGATAAAATAAAGTTCTCATTGTCCCCATCAAAATCAACGGATCGTTTGTCAACCAATGTCCCCGGAGTTCCTCTTGATGATAGAAATTTGATAATAAAGGCTCTCAATCTCTTCAGGAAAAAGACAGGGATTGACAACTACTTTTGGATTCATCTTGATAAGAAGGTGCCAACGGGAGCTGGCCTTGGTGGTGGAAGCAGCAATGCCGCTACTGCTTTGTGGGCAGCAAATCAGTTTAGTGGTTGTGTTGCTACTGAAAAGGATCTCCAAGAGTGGTCCGGTGAGATTGGCTCCGATATCCCCTTCTTTTTCTCACATGGTGCTGCATATTGCACGGGTAGAGGAGAGGTTGTTGAAGACATTCCTCCACCTGTACCTCTCGATCTACCTATGGTTCTCATTAAGCCACAAGAGGCATGCCCCACCGGTGAAGTTTACAAGCGTCTTCGGATGGATCAAACGAGCCAAATTGATCCTCTGGTGTTGCTAGAGAAGATAGCGAAGGGTGGAATCTCTCAGGACGTTTGTGTTAATGATCTTGAGCCTCCTGCTTTTGAAGTCGTTCCATCACTAAAAAGACTGAAACAGCGCATAGCTGCAGCAGGTAGAGGACAGTACGATGCGGTCTTCATGTCCGGAAGCGGGAGTACTATCATTGGAGTGGGTTCTCCTGATCCACCTCAGTTTGTTTACGATGATGAGGAGTACAAAAACGTCTTTTTATCAGAGGCCAAATTCATCACGCGGCCAGCTGATCAATGGTACTCGGAGCCTCTCTCAATCGATGAATCACCAAGCTTTTCTAAGGATGTTGAATAGTCGATATAATACCCATATATATATATATATATATATATATCATCTACAAATTCATTTGAATAAACATTGTGCTTGAAAAGTGCAAGTAACAATCACATTTTTAGTGTTGCTCTTTATTTGCTCCAATTTCATTGTATAATTTTTTTGTACTAGTTTGTAGTAATCAGAGTCCATAAGCAAAAAAAGAACACGCAACACAGCTGATATTAATTTGGGGGAAGAATAAAAGAAATAGAAGATGACATTAAAAGCAATGGAATTTGGATGCCTTTTTCTTTTTCCACTACTGAGATTGGCAATACACAATAAGAGAGAGGCATGTTCATTTCCTTGTCTTTGTATTTTTATTCTTTAGTTTCTCTTCTCCCCCTTCATCGTTTACAGCGAAGGGGAAGAAACAAACTGTCTGGGCAAACTGAAAAGGTCCTTCTCTTCAAATGGAGAGGTCCATTCCCGAGAGCCAGCCGCGTTCGGTTCCGTCGACTCCTGCATCGATGGGCCATTCCCACCCGATACAACCGGTCTGATCGCAGGCTTTACGCCCATTCCGTAACCAAATGAATCGAATGTACGAGTAGCATTGTTCTGTAAAGAGTAATTTGCACCATTCCACAACCCGTTCGGCCGGGAGCCAAGATCCAGGCTCCAGTCAATGTTGGTATAATCAAATTGTAACATTGACCCACCTGTGCTCCAGTCCACCGGAGATGAAGAGCCCGACGAGCCACTGGCGCCCAGCCCTGAGAAGAGCCCAAGGGACAAAGCAGCCGAAAGTGTTGGTGATGCACCTCCTCTGCTCCACATACCATTTCCTCTCGTGCTCGCCTGGGGATCCATTGAGCTAAACTGCGTCTGTGACTGGTAGTGCACTGGGTTGTACATTAGATTTGATCCAACACCACCCGGACTAATGCTTCTAGGTGGAACCAACCCATTGGGCTTCCCCACTTCAACATTGTTAGGATTAGGATACCAGCCCATGGCAGTCCCCGGAGGAGACGAGCTGACACTTGAGGTGGGAGCCTGCTTTGAGTTGACGGACTGAGGCCGCTGCATAACAATGACCGGTTCTCTCACTGACCCAAGCTGCAGATTCTTCAATTCATTCCCCACAGCGAGATAACGTGCTTCCATCTTGGCTGGAGGTGACGATGCCTGCAAAGGCGATGCTAATGAACAAGAGACCGAAGGATTGGATCCTGAACCAGGCCACCTTTTATCTGCAGGCCCTGCAATCTGCTGCGGCTTGCTCCAATCCATTTTAGGTGGAATCTTCTTCAATACCTGCATACTTTTCGTCCCAGGATCAATTGTCGGAGGCCCTACTGTCACTGGAACTTTGGTATAATTGAAATCTTTGTCATCCCTTTTTTGCTGTAAGATAGAAGATGAAGTCGGCTTGGCTTGCACCCTCATTGCATTGTGATGCATTGCAATACCTATCTTGCCATTGGAAATAGTTGGAGGATCACCAGTTTCTTCGGGCTTGGAAGGGACAGAAGGGGGCTCCTGTTTCTGCGTCTTCAACATTTCTTCAGCCTTCTCAAGGTCACCGTCAAAAGTTACAATAGCTCTCTCGACCTCCTGCTTTGATGCCTTATACCTCAATTCCATGTCAGTAATTCGTGCAAGCTCTTCTGATATGTCAATTTTCAAACTCCCCCCACCATCAAGATTTTGTTCCATCTGCTTGTCTTCTTCACCTCCTTCAAATAGCCATGCTACTGATTCTTCTACTCTACCTTCATTTAGTATGAGAGCCATTGTAGCCCGTTCTTGAGAGAAACCCATGGAAACAAGCTGTTGAGCAAGTGCTTCGAGCTTCCTCGACATGAGATAGCCACTGCACCGCTCATGTAGTTCTTGAGCTCGCCTCTCCTTTTGCCGCTGATGTTTCCTCTCATTTTTCTGGCGAATTTTTTCTCTTTTGTCATTGTCAGCTCCAGGTACTGAGTCCTGCCGTGATGTGGGCTGAGATGCCTTGTCCTTGTGGTCTTCTGACTCACCAGACCAGCTGCCATTATTGGAAACAGAGTCATACTCGACACCAGTCCCAAATGCATTCCCATTCTGATCATCCGTATCGTCTATGTTACGAAAACGGCCATTAACATGAAGTGGACTGACAGAAGGCACTGGTGGAGCCTCAAATGTATGGAATGTTCCCAAGAGAGGATTGTAACCACTTGATGGGATCCCTCCACTGGTGCTCACATGTCCTGAAGGCTTTATAGAACCCTTTGGAGGTTCCTTCCCAGCCTTTTTGTCTTTGGACTTAGATCTAGAAGCAGGAGACATGGCTCAACTTCAAGTTCTAATAGCTGCAACAAGGACAAGAGCTCCATTAGAACCAACTAAAGATAACACAAACAAAGCAAGTAAAATGGTACAGAGGGGACAAAATTTCTTTCCATATAGTAAATAAACAACTATAGTCAAAGTGGAAAATATGATGTTCATTCAAGAAGCGCAACCATGAAAGGTAGAGAAACATCAGAAAAATAAAGAGCAAAGACTAAACCCACTGATATATCATCCTGGATTCATAAGAAATAGTAAATAAATTGAAAGATTATTCAATGAGGAGGTCATGTGGCAGAATAATAAGTGTCTCCACCATGACCTCTATAAAACAAATGAAACTAACCATTCATCAAGTATCACAAGTAGATCTAAGTAGAAACTGAGAATGAAAAAAGTTACTTAAGTGTCAAAATGTGCATATGAAGGCTGAAGCTTAAAAGTAGAAACTGGTAAATAGTTGAAACCAATTGAACTTTCATAGAAATTAGTTTAACAGGAATAAGGTAGCTAACATGACATCATAAGAAACATGACTAATATCAGACACCATCCAGG

>SsHDS

GGCTACTAAAATGTGGAAAATGAAGATGCTGAGGAACTGAATTTTGTCTGTTTATTATCTTCAAATTGCTGTAGCTTTCATAATCTTTCGCCATTTCGGGCAACTATAAAACCTCTGAAGCCAGGCTCACTTTATTTCTGAATTTTCAGCTCCAAATAATTTCCATTTCAAATTCCTTCCACCACGAGTTCACCTCTCTCTCTCTCGATTCTTCCTTTCGGCTAATTGTTGCTACTCGTTTTTGTCATTTACCGAGGGGTGGAGTTCGGGACTTTGTGGAGTAAAATGGCGACTGGAGCTGTTCCGGCCTCGTTTACAGGTCTCAAAAGCAGGGATCGTCGTGGCTTGGGATTCGGAAAGAGCTCCGACTTTGTTAGAGTTTCTGACTTGCAGAGGGTTAAGTTTGGCAGGAGCAAGGTTGCAGTGATCAGAAACTCGAACAATCCCGGTTCGGAAACTGTTGAACTCAAGCCTGCATCAGAGGGAAGCCCACTGTTAGTTCCTAGGCAGAAGTATTGTGAATCCATACACAAGACTGTCCGGAGAAAAACCCGGACAGTGATGGTTGGAAATGTTGCTTTAGGTAGCGAGCATCCCATCAGAATTCAGACAATGACCACTACAGATACAAAGGATATTGCCGGGACTGTTGAGCAGGTCATGAGAATAGCAGATCAAGGAGCTGACCTTGTTAGAATTACGGTGCAAGGAAGGAAAGAAGCAGATGCATGCTTTGAAATCAAGAACACCCTTGTTCAGAAGAACTACAACATCCCTCTGGTGGCTGACATTCACTTTGCTCCACCTGTGGCTATGCGAGTTGCTGAATGTTTTGACAAAATTCGAGTTAACCCAGGAAACTTTGCTGATAGGAGGGCACAGTTTGAGATACTGGAGTACACAGATGATGACTATCAGAAAGAACTTGAGCATATTGAGAAGGTTTTCTCTCCTTTGGTTGAAAAATGTAAGAAGTATGGCCGGGCAATGCGCATTGGGACCAACCATGGTAGCCTTTCGGACCGCATTATGAGTTATTATGGAGACTCACCCAGGGGAATGGTCGAATCTGCATTTGAGTATGCAAGGATTTGTCGGAAGTTGGACTTCCACAATTTCGTGTTCTCAATGAAAGCGAGCAACCCAGTTATTATGGTTCAGGCTTACCGCCTTCTTGTAGCTGAAATGAATGTTCTAGGATGGGATTACCCATTACATCTGGGAGTGACTGAAGCTGGTGAGGGTGAAGATGGAAGGATGAAATCTGCGATAGGAATTGGGACACTTCTTACAGACGGTCTAGGTGATACCATTAGGGTTTCTCTAACTGAACCCCCAGAGGAGGAGATAGACCCCTGCAGAAGATTGGCTAATCTTGGAATGAGGACAGCTGAACTTCAGAAAGGAGTGGCGCCTTTTGAAGAAAAGCATAGACGCTATTTTGATTTCCAACGTAGAACTGGTCAACTGCCAGTCCAGAAAGAGGGTGAAGAGGTTGATTATAGAGGTGTGCTTCACCGTGATGGTTCAGTTCTGATGTCAGTTTCTCTGGATCAGCTGAAGTCTCCAGAATTGCTATACAAGTCTCTTGCAGCAAAACTTATTGTTGGAATGCCATTTAAGGATCTCGCAACTGTTGACTCAATCTTGCTGAGAGAACTTCCCTCCCAAGATGATAAAGATGCCAGGTTGGCTCTCAAGCGGCTGATAGACGTTAGCATGGGAGTAATAACTCCTCTATCGGAACAATTGACAAAACCATTACCCAATGCTATGGCCATGGTTACTCTCAAGGAATTGTCATCTGGTGCTCACCAGCTTCTTCCAGAAGGTACACGTTTGGTAGTTTCAATGCGTGGTGACGAACCCCTAGAAGAGCTAGAAATTCTGAAGACTACTGATGCTACCATGATCCTTCATCACATACCATATTCAGATGAGAAAACCAGCAGAGTTCATGCTGCAAGAAAGCTTTTTGAGTATCTATCGGAGAACTCCTTGGACTTCCCAGTTATTCATCATATAGAGTTCCCAAAAGGAATTCACAGAGACGACTTGGTTATCGGTGCTGGGACCAACGCAGGAGCCCTTCTGGTAGACGGACTAGGCGACGGTATCCTATTGGAATCCCCGGATCAAGAGTTTGAGTTCCTTAGAAACACTTCTTTCAATCTGCTACAAGGCTGCAGAATGAGAAATACAAAGACGGAATATGTGTCGTGCCCATCCTGTGGAAGGACGTTGTTCGATCTTCAAGAGATCAGTGCAGAAATAAGAGACAGGACGTCCCATTTGCCTGGTGTTTCGATCGCAATCATGGGCTGCATTGTAAATGGCCCCGGAGAAATGGCTGACGCAGATTTTGGCTACGTTGGTGGCGCCCCTGGAAAGATCGACCTTTATGTTGGCAAGACGGTGGTGAAGAGAGGTATCGCGATGGAGCATGCAACGGATGCGCTGATCGAGCTGATCAAGGAGCACGGCCGATGGGTGGATCCACCAGTTGAGGAGTGAAGGTGATGGCTTCTGTAGCATTGTATCTCCAAAGAAGATCATCTGCTGCTCAAAGCAATACTAGTGGATGAGAATGTGGTGATATATTTATTACATAAATTTTTTTGTTTTCTTTTTCCCCTTCAAATTTGATGATAAGTAATGGAGTTCCTTTCCCTGTTGTTGGAACTTGAAAGAAGATCCATTTGTATGACTACAGAGTAAGTGAATGAATGCAAGAGAATGATTTTAGCACTGCCCAATCATAATATGTAGGCTGTTGTATATATTAATTGTAGTTAAAAGTACTCACCCATTTAAAATATAAAATAATAATAATAATGGACATATTCTCACCTCCCAAATGAAGGCTCATTTTTGCAAACAATGAAATTTAAGATT

>SsHDR

AAAAGTTTCAATTCTTTTGACCATTGCATGCTTGCTGCTTCAGCAGTCAAACATTGAACAAAAAAATAATTTTGAGCGAAGAAATTGGAGAATCAAACATCCTATAGTGGAATCATAAATATAGGAGGGCGACAAATGCAAGGAAAATCAAATCTATTAATAATAAAGAAAAATCATATATAAATTTGATTTGGCGAAGAATGCGTCAGCAATAATCAGATTAAATAAACAAGGCAAAAGATAAATTGCCGCAAATCCAGTCCAGCTCTGCTTTGTAAGAAACCACACTGATAATTCATTCGCATGGCGATCTCTCTGCAATTCTGCCGCATCTCAACGCGCACTCAAATCCCCTTGCCGGAGACCAGGTTTCCCCGGCGACTGGGGCCTCCCTCCGTCCGGTGCTCCGCTGCCGGAGATGGCGCCGCCTCATCCTCTTCCGTGGCCGCTGATTCCGCCGAGTTTGACTCCAAGGTTTTCCGTCACAACCTCACCAGGAGTAAGAATTACAACCGGAAGGGGTTCGGGCATAAAGAGGAGACGCTCATGCAAATGAGCCAAGAGTTTACGAGTGACATTATTAAGAAATTGAAGGACAACGGTTATCAATACACATGGGGGGATGTTACGGTGAAGCTTGCTGAAGCTTATGGATTTTGCTGGGGGGTCGAGCGTGCGGTTCAGATTGCTTATGAAGCTAGAAAACAGTTCCCGTCTGAGAACATCTGGCTGACTAATGAAGTTATCCATAACCCCACGGTTAATGGGCGGCTGGAAGAGATGAATGTAAAAACTATTCCAAATATCGATGGGAAGAAAGAATTCGATGTTGTTGACAAAGGTGATGTTGTGGTATTGCCTGCTTTTGGAGCTGCTGTTGATGAGATGCGGATTTTGAGCGACAAGAATGTTCAAATAGTTGATACAACTTGCCCATGGGTGTCTAAGGTTTGGAATACTGTTGAAAAGCACAAGAAAGGAGAGTATACTTCTATAATCCATGGTAAATATTCCCATGAAGAGACTGTGGCCACTGCTTCTTTTGCGGGGAAATTTGTTATTGTAAAGAACATGCAAGAGGCAACATATGTATGTGACTACATCTTGGGTGGTGGACTTGATGGATCCAGCTCTACTAAAGAAGCATTTCTCGAGAAATTTAAATTGGCAATATCTAATGGATTTGACCCCGACAAGGATTTTGAGAAAGTTGGTATTGCGAATCAAACTACTATGTTAAAAGGAGAAACAGAGGATATTGGAAAATTAGTCGAGAGGACCATTATGCGCAAGTATGGGGTTGAAAATGCTACCGACCACTTCATTAGTTTCAACACTATTTGTGATGCAACTCAAGAAAGGCAAGATGCAATGTATAAATTGGTCGACGATCCGGTGGATCTCATCCTAGTCATTGGCGGATGGAACTCAAGTAACACTTCACACCTACAAGAAATTTCAGAATTGCGTGGAATTCCATCCTATTGGGTTGACAGTGAGAAGAGGATAGGTCCTGGAAACAAAATTAGTCACAAGCTGATGCATGGCGAGTTAGTGGAGAAAGAGAACTTTCTACCAGAGGGTCCTATCACAATTGGCGTTACATCCGGTGCATCTACTCCCGACAAGGTTGTTGAAGATGTTCTTCAAAAGATTTTCGAGTTAAAGCACTAGGATGTAATGAAATCAGCTTAAGCTCTGTGTCATGATTCAACATTGCATTGATTAGTTCAAATCCGTCTGTGGATTAAGCAAGGGTAGCCGCCTTGTTTCCTTGCTGTGTAAACTATATGTAGCTGTATGTATGAATGTCGCGTAAACTGAGATTCCGTTATGCAGACTCGCGCTCTCTGTAAGGAAAGTATGCGCGAATCCAACATATTACGCTTTGATAATAAGGAAAATTTTAGTTTTGCGATGTATGAGAAGTGATCCGACTCTGTGGCTGTAAATGCAAGGGTTTTGTTCAAAGTTCCAACAGAACTCTGTGCTTGAATAATAACAAAATCTGTTGGTTATGCTCCACTCTCAACTCATTTTTCTAAAAATTATAAATATTGATATGCTGTATATTTAGATATAGTAATAATATATTGTAGATGACAAATATGCAGCACACTGCACACTACCATGAAAGTCGATTTTGGA

>SsGGPPS

GTGTATTTTATGCGAGGAGTGGGGCACCGATTTAGTGAAGCAAACACAATTATAACCTCAGTAATATTTATTTGAGAATAATGGGATTTTAGAGTAGAATCCAAAAGTGGTTTCTATGCATTTTTGACTGCCCCTCATCCAAAACCCTATCTCTTCTCTCTCACATACACACTAGTATATATGTGTGCGCATTTCTCTCGAATCAAACTCCATCAACAGATTTACCCGAACTCTTCTTAGCTCAAAATCCAAAATTTACAAATCATGAGGTCTATGAATCTGATGGATGCCTGGGTCCAGAACCTCTCAATCTTCAAGCAGCCTCACCCCTCCAAATCCCTGCTCGGATTCATCAACCACCCCAGATTCGAACCTGTTTTCCTGAAATCACGCAAGGCCATCGGCGTCTCCGCCGTCCTGACCGGCGAGGAGGCCAGAATCTCGACTCAGCCCTTCAATTTCAACGCCTACGTGGTGGAGAAGGCGAATCACGTCAACAAGGCGCTGGACGAGGCGGTGGCGGTGAGAAACCCGCCGATGATCCACGAGGCGATGCGCTACTCGCTACTCGCCGGCGGGAAGCGCGTGCGCCCCATGCTCTGCATCGCCGCCTGCGAGATCGTCGGCGGACCCCAGGCGGCGGCAATCCCGGCCGCCTGCGCGGTGGAGATGATCCACACCATGTCGCTCATCCACGACGATCTCCCCTGTATGGACAATGACGACCTCCGCCGCGGCAAGCCTACCAACCACAAGGTCTTCGGCGAGGATGTCGCCGTGCTTGCAGGGGATGCTCTGTTGGCCTTCGCTTTCGAATTCATGGCGACGGCGACGGAGGAGGTGGCGCCGGAGAGGATACTAGCGGCGGTGGGGGAGCTGGCGAAGGCGATCGGGACGGAGGGGCTGGTGGCTGGGCAGGTGGTGGACATCAACTGCACCGGCGACGCAAATGTAGGATTAGACACATTGGAATTCATCCACATACACAAAACTGCTGCATTGCTAGAGGCTTCTGTAGTTTTGGGGGCCATTTTGGGGGGTGGCAGCAGCGATCAAGTTGAGAAATTAAGAACTTTTGCTAGGAAAATCGGCTTGCTTTTCCAAGTTGTGGATGACATTTTGGATGTGACCAAATCGTCGGAGGAGTTGGGGAAGACGGCCGGGAAGGACTTGGTCGTGGACAAGACCACGTATCCGAAGCTGCTGGGGCTCGAGAAGGCCGTGGAGTTTGCCGAGAAGCTCAACGAGGAGGCCAAGGCGCAGCTGGCTGAGTTCGACCCGGACAAGGCGGCACCGCTGGCAGCGCTGGCCGATTACATTGCTCATAGGCAGAACTAGTTATGCTGCTATATGGAAATCTAGTGTTTGTTGTAGAAAAAAAAGAAAAAAGATATTCTGCTATGCAATGAAATAGCATAGCAATTCTTTTGGTATACCATTGGATGTCAACTTATATGGAAACTTGTTTCTTCATCATGTCATGATCTTGCCTTGGTGTCTACTAATCTCTTTTTTCTTGATTATTATGTAGATTTTGTACATTATGAATTTGAATGTAATAATGGTAAAAGGTAAAGTTTCTGATCATCAAAAGCTTTGTTTATACTTGGTTGTGGAGTTCTTGATTGTGGAAA

>SsRbcS

ATGGCAGCCTCAATGGTCTCCACCGCCGCCGTGGCCGCCCGCTCGGCCCCCGCTCAGGCCAGCATGGTCGCCCCCTTCACCGGGCTCAAGTCCGTCTCCGCCTTCCCCGCCACCCGCAAGACCGCCGACATCACCACCATCGCCAACAACGGTGGCAGAGTCTCCTGCATGAAGGTGTGGCCCACCGAGGGATTGAAGAAGTTCGAGACCTTGTCGTACCTTCCCCCTCTAACCAGGGAGCAGCTCTTCAAGGAAGTCGAATTCCTTATCCGCACCAACCTCATTCCTTGCCTCGAGTTCGAATTGAAGGAAAAGGATGGATTCCCACACCGTGAGAACAACAGGTCCCCCGGATACTATGACGGAAGATACTGGACAATGTGGAAGCTGCCCATGTTCGGGTGCACTGACCCCGTGCAGGTGCTCAAGGAGTTGGACGAGTGCTCCGACCTCTACCCACAGGCCTTCATCAGAATCATCGGATTCGACAACAAGCGTCAGGTGCAGATCTGCAGTTTCATTGCCCACAGGCCGCCAGGCTAC

>SsCAB

ATGGCAGCCAACACCTTGATGAGCTGCGGCGTCGCCGCCGCCGCCATCTGCCCCTCCGTCCTCTCCTCCTCCAAGTCCAAATTCGCCGCCTCCGTCTCCTTCGGCACCAATGCCACCACCTCCAGGTTCTCCATGTCCGCCGAGTGGATGCCGGGCGAGCCCCGCCCACCCTACCTAGACGGCTCAGCTCCGGGAGATTTCGGATTCGACCCACTTCGCCTAGGGGAAGTCCCAGAAAACCTAGAGAGATATAAGGAATCGGAGCTCATCCACTGCAGATGGGCTATGCTCGCCGTCCCCGGGATCCTGGTGCCAGAGGCTTTGGGCCTGGGCAACTGGGTCAAGGCGCAGGAGTGGGCTGCACTGCCGGGTGGGCAGGCGACCTACTTGGGCAACCCGGTGCCATGGGGCACCCTTCCCACCATCTTGGTGATCGAGTTCTTGTCCATAGCCTTTGTAGAGCACCAAAGGAGCATGGAGAAGGATCCGGAGAAGAAGAAGTACCCGGGCGGGGCATTCGACCCTCTCGGATACTCCAAGGACCCCAAGAAGTTCGAGGAGCTCAAAGTCAAGGAGATCAAGAATGGTCGTCTTGCCCTGCTGGCATTCGTGGGATTCTGCGTGCAGCAATCAGCGTACCCGGGAACCGGACCATTGGAGAATTTGGCGTCGCATTTGGCTGACCCGTGGCACAACAACATTGGCGATGTCCTAATTCCTCTTTCC

>SsGAPDH

CCAAATTCTCCCACAAACACAAAGTGGTCCATAACGGCTCATACACCAGGCGCACTGTATAACTCCGCCTCGTGCCTGCTTATCTACAATACTAACTTAAACCCATCGCACCTCTTCTTCTCACTTTCCACGCACATCTTCTCCCTCACTAAAAACTCTTGCTCTACCACTCGACTTTCCTCGCTCTCTCTCACTCTACCAACTACAAATGGCGAAGATTAAGATCGGAATCAATGGTTTTGGTAGAATTGGCCGTCTGGTTGCGAGGGTTGCTCTTCAAAGAGACGATGTTGAGCTTGTTGCTGTCAACGATCCGTTCATTACCGTTGACTACATGACCTATATGTTCAAGTATGACAGTGTGCATGGCCAATGGAAGCACCATGAGCTTAAAGTTAAGGATGATAAAACCCTTCTCTTCGGTGAAAAGCCTGTGACTGTTTTTGGCTTTAGAAATCCTGAGGAGATTCCATGGGCTTCGACTGGAGCTGAGTACATTGTGGAGTCAACTGGTGTTTTCACTGACAAGGACAAGGCTGCAGCTCATTTGAAGGGTGGCGCGAAGAAGGTCATTATATCTGCTCCAAGCAAGGATGCACCCATGTTTGTCGTTGGTGTCAATGAGAAGTCATACACACCAGACCTCAACATTGTTTCTAATGCTAGCTGCACCACAAACTGCCTCGCCCCATTGGCAAAGGTCATTAATGATAGGTTTGGTATTGTTGAAGGCCTTATGACAACTGTCCACTCTATCACTGCGACCCAAAAGACTGTTGATGGACCATCTGCCAAGGACTGGAGAGGTGGAAGAGCTGCATCATTCAATATTATCCCAAGTAGCACTGGAGCAGCTAAGGCTGTTGGCAAAGTTCTCCCAGCTTTGAATGGAAAATTGACCGGAATGGCATTCCGTGTCCCAACAGTTGATGTTTCCGTGGTTGATCTTACAGTGAGGTTAGAGAAGGAAGCTACTTATGATGAAATCAAAGCAGCGATCAAGGAGGAATCTGAGGGAAAGATGAAGGGAATTCTAGGCTACACTGAAGACGATGTGGTGTCTACTGACTTTGTCGGTGACAACAGGTCGAGCATTTTTGATGCCAAGGCTGGAATTGCTTTGAGTAAGAACTTTGTCAAGCTTGTTTCGTGGTATGACAATGAATGGGGTTACAGCACTCGTGTGGTTGATCTGATCAAGCACATTGCCTCCACTCAGTAACTTTCTACGGCTTGCTGTATTCTTCTAGGGTACCACATCTGTTGTGGTGTTTTGGTGGAACTTGAGTTGGAATAAATTGTTGTCTGTTTGAGACTAAGTTTTAGATGTATGGCTTGTTCTTATGAGTGCATAATTCTGGCAGATTGGTGGGGTAGTGACTACTCCTGTATTATGAAGAATTTTGCTTTGAATGGTGCATGTTAGATATCTTTATGTGTACTAATTTGCATTAGAACCATCGGTTTCTGACCTTTGCTTGTTGAGGGTCAATCCTTGAATAATAGGTGAACTGTTGCTGCTTTATGTTAACTCTTGTGTGACTGAAAAGGTGTCTTTAGGGATTGTATGGCCATTTGTGTTGGAGAGATCCAACTTTTTGGTATCATGTCGCAGTCTACTTTTATTGATGGTGGTTGGACGGCAGGCAAATTATCTTTTCAGGATTTCGCAGTATCTAACTTTCTTGTTATTTTGGTGGTTTGCTGGCCAGGTTTACTCATGTGAGTATCTGTGGGGCTTGAGTTTGTATATGTAGTCTTATCCAGTTTTTGCTTCTAAAATCGATTTTTCTCCTATTATCAGTGGCGAATGGGATATATGTGAACAAGGGTTATTTTTTTTCTAACGGTAATCCAAATTTTAACCCATGTTAATTTTTAGCTTGTGTTCTATAAATAGGCAAAATCTCCTATACTCTATATACACATTACCTTGTAATCAAGGGTGATGGGAAGGTAGCAAAGGAATAAATTTTGAATTTGAATAATGATGTATGTATATACTGATGTATAGGAGTCTACCTCGTATAAATATTATGAGTACAGCTTGAACT

>SsACTIN

GGAAAAGGCAGCCAGCTACATTGACCATCAATGTAATTGCTGTCGTGGGTGCCACAACCCACAAATCCAAATCATTTCAATCTAACGGCTGAGATCTCGTAATCTTGAATTTGAGGTTCATTTGATCTGAATTTAATGTGAGGCTGAGGTTTCATTTCTCGTATTTTAAACGAGAGGGCCGGCATTTCATTTCCAGACCAACACGACACAGACAGAGTCCACATTATCTCTCACAGAGTCTCTCGGACCCCAACTTCTTCGGCAACTCCTCAGAGATAACATTAATCGATGGCAGACACGGAGGATATTCAGCCCCTCGTTTGCGACAATGGAACTGGAATGGTCAAGGCTGGATTTGCTGGGGATGATGCTCCGAGAGCTGTGTTTCCAAGTATAGTGGGGCGCCCTCGCCACACTGGAGTTATGGTTGGCATGGGCCAGAAGGATGCTTATGTCGGTGACGAGGCTCAATCCAAGAGAGGTATTTTGACGCTGAAATATCCGATTGAGCATGGAATTGTGAGCAACTGGGATGATATGGAGAAGATTTGGCATCATACCTTTTACAATGAGCTTCGTGTGGCCCCAGAAGAGCACCCGATCCTCTTGACCGAAGCTCCTCTCAACCCCAAGGCCAATCGTGAAAAGATGACCCAAATCATGTTTGAGACCTTTAACACCCCTGCTATGTATGTCGCCATTCAGGCTGTTCTCTCTCTGTATGCTAGCGGTCGTACCACTGGTATTGTTCTCGACTCTGGAGATGGTGTCAGCCATACAGTTCCAATCTACGAGGGTTATGCTCTCCCCCATGCGATCCTCCGTCTCGATCTTGCTGGCCGTGATCTCACCGACAGCCTTATGAAGATCCTCACAGAGCGTGGCTACATGTTCACAACTACAGCCGAGCGAGAAATCGTGAGGGACATCAAGGAGAAGCTAGCTTACATTGCTCTCGATTATGAACAGGAGCTTGAGACTGCAAAGACAAGCTCTGCTGTGGAGAAGAACTATGAGCTGCCTGATGGACAGGTCATCACTATCGGAGCTGAGCGGTTCAGATGCCCTGAGGTCCTCTTCCAGCCCTCGATGATCGGGATGGAAGCTGCTGGTATTCACGAGACCACCTACAACTCCATCATGAAGTGTGACGTCGATATCAGGAAGGATCTGTATGGAAACATTGTCCTCAGTGGTGGCTCGACTATGTTCCCGGGTATTGCTGACCGGATGAGCAAGGAGATCACAGCTCTTGCCCCGAGCAGCATGAAGATCAAGGTGGTGGCCCCACCGGAGAGGAAGTACAGTGTCTGGATTGGAGGGTCCATTCTGGCTTCTCTCAGCACATTCCAGCAGATGTGGATTGCCAAGGCGGAATACGACGAGTCCGGCCCTTCGATCGTGCACAGAAAATGCTTCTAAAAATCGAACTCTTCGTTCGCTGCTTTGCTTGAGGAGGCTATACATGATATGATATGCCCTATGCTCTCTGCTTCATTCGCTTTTCTTCCGGTTTTGTTCGTTTATCGAGTCGTCTTTGCCTATGTTCTGAGAATTTGAAGTTTGGACATGGAAATATTTTCATTTTTTTTGGTTCCATATTTATATTAGTTTGTAGCAGACTTGTGCTTTTCCGGTGGTACTATGGTTTGAGTTTTGGATGAAGAGCACATACTGTAATTTTAGTATGGAATTCGTGATGTCAAGATTCAATCTTTGCTTTTCTCGTGATTTTGTTTTTTAAGAATATATTGTTTGGTAAATTGCTATAAATTTCTCGTTGTTTGACCCGATTTAGTAGTCTGCAGGTTTTAGGCCATCTCCAGCGCTGCGCTTTTATTGCGCTCAGCGTTATTTTGCAGCCAATTTCCACTCCCAGCGCTGCGTTATTTCTTTGCGCTATTTTTGAGAAAACAGTAATCATACGCCATTTTTGTGTGGAGCTTGAGCTGCGCTATCCATGTTTTTCCTTTTCCTTTTCCATGTTTTTCCTTTTCCTTTCCTTTTCCTTTTCCTTTTCCTTTTCCTACTTTTTAATAAGTAGTATTAATGTATAGTATATTTTTGTGTGTACATTATATTGATTTAAAATGGAAGGTTATTAGTGCATAA
